# Identification and Biosynthesis of a Novel Xanthomonadin-Dialkylresorcinol-Hybrid from *Azoarcus* sp. BH72

**DOI:** 10.1371/journal.pone.0090922

**Published:** 2014-03-11

**Authors:** Tim A. Schöner, Sebastian W. Fuchs, Barbara Reinhold-Hurek, Helge B. Bode

**Affiliations:** 1 Merck Stiftungsprofessur für Molekulare Biotechnologie, Fachbereich Biowissenschaften, Goethe Universität Frankfurt, Frankfurt am Main, Germany; 2 Lab of General Microbiology, University of Bremen, Bremen, Germany; University of Florida, United States of America

## Abstract

A novel xanthomonadin-dialkylresorcinol hybrid named arcuflavin was identified in *Azoarcus* sp. BH72 by a combination of feeding experiments, HPLC-MS and MALDI-MS and gene clusters encoding the biosynthesis of this non-isoprenoid aryl-polyene containing pigment are reported. A chorismate-utilizing enzyme from the XanB2-type producing 3- and 4-hydroxybenzoic acid and an AMP-ligase encoded by these gene clusters were characterized, that might perform the first two steps of the polyene biosynthesis. Furthermore, a detailed analysis of the already known or novel biosynthesis gene clusters involved in the biosynthesis of polyene containing pigments like arcuflavin, flexirubin and xanthomonadin revealed the presence of similar gene clusters in a wide range of bacterial taxa, suggesting that polyene and polyene-dialkylresorcinol pigments are more widespread than previously realized.

## Introduction

**Figure 1:**
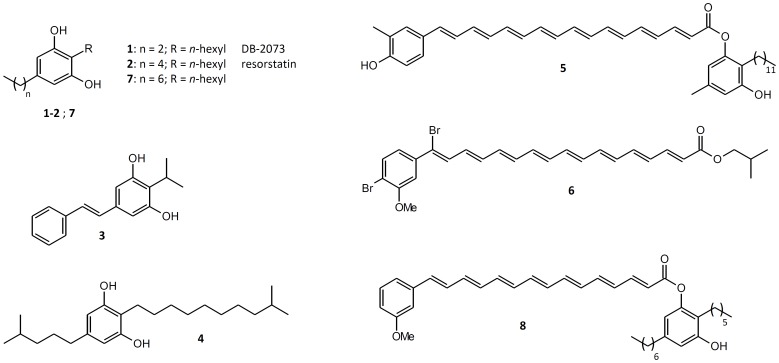
Natural occurring DARs (1–4) [2]–[6], polyene containing pigments like flexirubin (5) from *Chitinophaga filiformes* [8] or xanthomonadin I (6) [9], DAR (7) heterologously produced with *Azoarcus* DAR proteins [1] and the proposed structure of the DAR-xanthomonadin hybrid pigment named arcuflavin (8) from *Azoarcus* sp. BH72 identified in this study.

**Figure 2:**
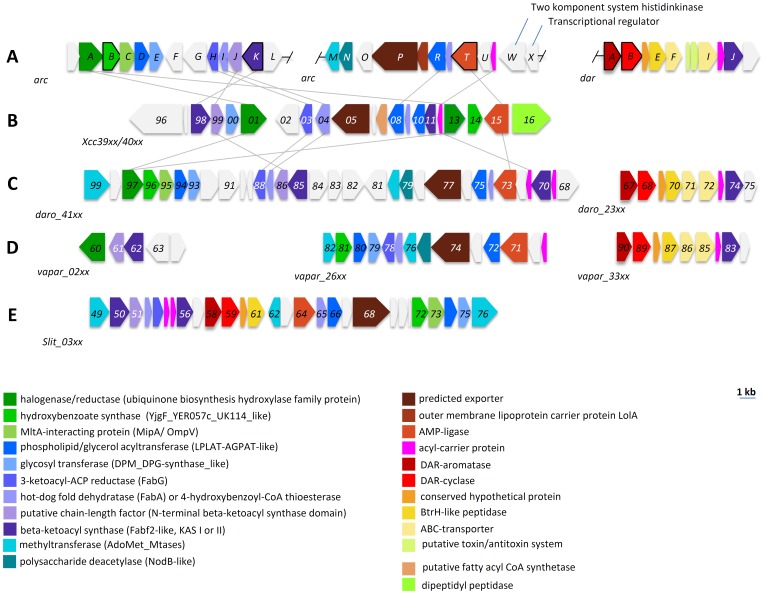
Known and proposed gene clusters involved in biosynthesis of DARs, xanthomonadin and xanthomonadin-like compounds in *Azoarcus* sp. BH72 (A), *Xanthomonas campestris pv campestris* ATCC 33913 (B), *Dechloromonas aromatica* RCB (C) *Variovorax paradoxus* S110 (D) and *Sideroxydans lithotrophicus* ES-1 (E). Annotation colors follow the *Azoarcus* gene clusters and genes are connected by grey lines if their identity was ≥40% in a BLAST-P analysis. All genes are scaled to the depicted size and are listed in Table S1, S2, S3, S4.

An underexplored class of natural compounds are the 2,5-dialkylresorcinols (DAR), microbial secondary metabolites, which are derived from a condensation of two fatty acid metabolism intermediates [1]. DAR examples with known bioactivities (Figure 1) are the free radical scavengers DB-2073 (**1**) [2]–[4], resorstatin (**2**) [4], a multipotent isopropylstilbene (**3**) [5] or the mammalian cell growth stimulating factor resorcinin (**4**) [6] which can also be found as part of the flexirubins from *Flavobacterium johnsoniae* [7]. Flexirubins (**5**) [8] are orange pigments made of a DAR esterified with a non-isoprenoid aryl-polyene carboxylic acid and are used as chemotaxonomic marker for bacteria from the *Bacteroidetes* phylum. Another class of aryl-polyene pigments, the xanthomonadins, has been found in bacteria of the genus *Xanthomonas*. Besides the dibrominated xanthomonadin I (**6**) [9], derivatives with different levels of bromination, methylation and chain length are known [10], all lacking a DAR-moiety. The gene cluster for xanthomonadin biosynthesis (Figure 2.B) is conserved in several strains of *Xanthomonas* [11]–[13] and was recently studied in *Lysobacter enzymogenes* [14], another bacterium from the family *xanthomonadaceae*. The gene cluster encodes an acyltransferase, a ketoreductase, a dehydratase, a predicted ketosynthase, an unusual chain-length factor like protein [14] as well as a chorismate-utilizing enzyme [15]–[17] suggesting, a type II fatty acid synthase like biosynthesis of xanthomonadin. Whereas the biological role of flexirubin is unknown, it could be shown that xanthomonadins protect *Xanthomonas* against photooxidative damage and lipidperoxidation [18]–[20]. Similar results were obtained for the xanthomonadin-like pigment from *L. enzymogenes*, which protects the bacterium from UV/visible-light and H_2_O_2_ [14].

Recently we reported that DAR biosynthesis is encoded by a conserved gene cluster which was found in 89 bacterial strains with most of them being associated with other organisms [1]. Among them is *Azoarcus* sp. BH72, a rod shaped motile bacterium which was isolated from surface-sterilized roots of Kallar grass (*Leptochloa fusca*) [21] where it lives as diazotroph mutualistic endophyte. Since heterologous expressed DAR-biosynthesis proteins of *Azoarcus* sp. BH72 led to DAR (**7**) (Figure 1) production [1] but no DAR was detected in *Azoarcus* sp. BH72 extracts, we were interested about its fate in the bacterial secondary metabolism. As it is known that *Azoarcus* sp. BH72 produces a cell-bound yellow pigment [21] we speculated that its DAR might be connected to the yellow compound in a flexirubin-like manner.

Here we report gene clusters responsible for the pigment biosynthesis in *Azoarcus* sp. BH72 and show that gene clusters containing polyene-biosynthesis associated genes are widespread in bacteria. Furthermore, we show by a combination of labeling experiments, HPLC-MS and MALDI-MS that the pigment from *Azoarcus* sp. BH72 contains the already reported DAR **7**, which is connected to a xanthomonadin-like polyene. Additionally, a benzoate synthase and an AMP-ligase encoded by the *Azoarcus* pigment biosynthesis gene clusters were characterized that might perform the first two reactions of the polyene assembly.

## Materials and Methods

### Cultivation of strains

*E. coli* and *Azoarcus* sp. BH72 strains were cultivated on solid LB-medium (per L 0.5% yeast extract, 1% tryptone, 0.5% NaCl, 1.5% agar) or in liquid LB-medium on a rotary shaker at 200 rpm and are listed in Table S5. For protein overproduction *E. coli* was grown in modified auto induction medium (per L 0.5% yeast extract, 1% tryptone, 0.5% NaCl, 50 mM Na_2_HPO_4_, 50 mM KH_2_PO_4_, 25 mM (NH_4_)_2_SO_4_, 0.5% glycerol, 0.05% glucose, 0.2% lactose, pH 6.75) [22]. For extraction of chromosomal DNA and labeling experiments *Azoarcus* sp. BH72 was cultivated at 30°C and *E. coli* strains at 37°C. Where appropriate, kanamycin (50 µg/mL) was added to the medium.

### Genome mining

For the identification of DarAB biosynthesis gene clusters, the primary sequences of the DarAB homologues from *C. pinensis* DSM 2588 were used for a BLAST-P identification (protein-protein-BLAST) of homologues in *Azoarcus* sp. BH72, *Variovorax paradoxus* S110, *Dechloromonas aromatica* RCB and *Sideroxydans lithotrophicus* ES-1. The xanthomonadin gene cluster from *Xanthomonas campestris* pv. *campestris* ATCC 33913 was used in a STRING-analysis (version 9.0) [23] for the identification of the putative xanthomonadin polyene cluster in *Azoarcus* sp. BH72, *V. paradoxus* S110, *D. aromatica* RCB and *S. lithotropicus* ES-1. Gene colors in Figure 2 are based on NCBI-annotation or domain guided annotations from BLAST-P with primary sequences of *X. campestris* pv. *campestris*-genes as template (see Table S1, S2, S3, S4). Grey connections between genes highlight BLAST-P results with an identity ≥40%.

### Feeding experiments

400 mL liquid LB-medium was inoculated 1∶100 from an overnight culture of *Azoarcus* sp. BH72. After 3 h L-[*methyl*-^2^H_3_]methionine or 4-fluoro-3-hydroxybenzoic acid was fed in 1 mM portions to the cultures and feeding was repeated after 12, 24 and 48 h, resulting in a final concentration of 4 mM. As control, a culture without feeding was cultivated at the same time. Due to poor growth compared to the control culture, feeding of *Azoarcus* sp. BH72 with 4-fluoro-3-hydroxybenzoic acid was stopped after 24 h at a final concentration of 2 mM and cultures were grown for additional 48 h before harvesting. Cultures were harvested 12 h after the last feeding by centrifugation at 10.000 rpm.

### Extraction and isolation of compounds

Pelleted cells of liquid cultures were shaken for 30 min with 50 mL acetone in the dark, followed by filtration to remove cell debris. The extracts were dried under reduced pressure. For structure elucidation raw extracts were fractionated using silica gel columns with hexane/ethylacetate (4∶1; 1% acetic acid). Yellow fractions were dried under reduced pressure. Samples were stored dry at −20°C in the dark and dissolved in 10–50 µL acetonitrile (ACN) before further analysis.

### HPLC-MS

ESI HPLC MS analysis was performed with a Dionex UltiMate 3000 system coupled to a Bruker AmaZon X mass spectrometer and an Acquity UPLC BEH C18 1.7 µm RP column (Waters) as described previously [24].

### MALDI-MS

For structure elucidation MALDI-analysis, samples were mixed 1∶2 with 1 µl of a 20 mM 4-chloro-α-cyanocinnamic acid (ClCCA) in 70% ACN with 0.1% trifluoracetic acid (TFA) or 9-aminoacridin in acetone, spotted onto a polished stainless steel target and air-dried. MALDI-MS analysis was performed with a MALDI LTQ Orbitrap XL (Thermo Fisher Scientific, Inc., Waltham, MA) equipped with a nitrogen laser at 337 nm as described previously [25]–[26]. Qual Browser (version 2.0.7; Thermo Fisher Scientific, Inc., Waltham, MA) was used for spectra analysis and to calculate possible sum formulas.

### Molecular-biological methods

Molecular-biological experiments were performed according to standard procedures [27]. The phusion high-fidelity polymerase (Thermo-scientific) was used for polymerase chain reactions (PCR) according to the manufacturer's instructions and with oligonucleotides (Table S6) obtained from Sigma-Aldrich. Plasmid isolation was performed with the GeneJet™ Plasmid Extraction Kit (Fermentas) and DNA extraction from agarose gels with the GeneJet™ Gel Extraction Kit (Fermentas).

### Generation of mutants of *Azoarcus* sp. strain BH72

Genes *azo3911 (arcK)* and *azo0260* (*arcT*) were disrupted via plasmid integration with plasmid pK18GGST [28]. Briefly, internal gene fragments of the 5′ region of the respective genes were amplified and cloned into the expression vector pK18GGST through specific restriction sites, and a single recombination event with the wild type chromosome resulted in plasmid integration and thus a polar mutation. Growth conditions for mutant generation were as previously described [29]. The primers used for amplification were for *azo0260* azo0260KOFor (*Xba*I restriction site) and azo0260KORev (*Hin*dIII restriction site), and for *azo3911* azo3911KOFor (*Xba*I restriction site) and azo3911KORev (*Hin*dIII restriction site), respectively. The resulting fragments 0260KOfragment and 3911KOfragment cloned into the *Xba*I-*Hin*dIII restriction sites of pK18GGST spanned nucleotide positions of 7–639 nt of gene *azo0260*, or of 16–625 nt of gene *azo3911*, respectively. The correct plasmid sequences were verified by sequencing. Plasmids were transferred to strain BH72 by biparental mating, and the resulting plasmid integration mutants BH0260 and BH3911 were selected by their kanamycin resistance. Integration of the plasmid into the correct target site in the chromosome was verified by Southern blot hybridization.

### Construction of plasmids for heterologous expression of ArcB and ArcT

An expression cassette encoding the TEV-protease cleavage site and a cherrytag (DelphiGenetics SA) with c-terminal His_6_-tag was digested with the restriction endonucleases *Nco*I and *Avr*II and ligated into the similar treated pCOLA-DUET1 resulting in pCATI1. The genes *arcB* (*azo3920*) and arcT (*azo0260*) were amplified by PCR from genomic DNA isolated from *Azoarcus* sp. BH72, resulting in 3920fragment and 0260fragment respectively. For construction of pCATI-arcB, pCATI1 was linearized by PCR giving pCATI1fragment which was fused with azo3920fragment using the Gibson assembly cloning kit (NEB). For construction of pCATI-arcT, 0260fragment was digested with the restriction endonucleases *Nco*I *and Xho*I and ligated with the similar treated pCATI1. The inserts of the plasmids pCATI-arcB and pCATI-arcT were sequenced by SeqIT GmbH (Kaiserslautern).

### Overproduction and purification of ArcB and ArcT

*E. coli* BL21 (DE3) Star cells were transformed with pCATI-arcB or pCATI-arcT resulting in strain TS3920 and TS0260, respectively. 200 mL auto induction medium was inoculated 1∶100 from an overnight culture of TS3920 or TS0260 and grown to a OD_600_ of 0.6–0.8 at 37°C, followed by 16 h at 18°C. After centrifugation, the cell pellets were frozen at −20°C. Cells were lysed by sonification in lysis buffer (500 mM NaCl, 20 mM imidazole, 20 mM tris(hydroxymethyl)aminomethane (Tris), 0.01% Triton X-100, pH 7,5) which additionally contained lysozyme (10 kU/mL) and benzonase (2,5 U/ml). After filtration of the soluble fraction through 0.6 µM syringe filters it was applied to a ÄKTAexplorer™-System (GE-Healthcare) equipped with a HisTrap™ HP 1 mL affinity-chromatography column. Binding and elution buffer contained 500 mM NaCl, 20 mM imidazole, 20 mM HEPES pH 7,5 and 500 mM NaCl, 500 mM imidazole, 20 mM HEPES pH 7,5, respectively. Fusion tags were cleaved with TEV-Protease in elution buffer (12 h, room temperature) and tag-less proteins were purified as mentioned above. Purified proteins were analyzed by SDS-PAGE, protein concentrations were determined on a Nanovue Plus Spectrophotometer and proteins were stored at −80°C in storage buffer (100 mM NaCl, 50 mM Tris (pH 7,5), 1 mM dithiothreitol, 1 mM EDTA, 10% glycerol).

### Benzoate synthase activity assays with ArcB

For *in vivo* activity assays with ArcB, 1 mL auto induction medium was inoculated 1∶100 from pre-cultures of *E. coli* BL21 (DE3) Star containing pCATI-arcB (strain TS3920) or the empty vector (pCATI1). After incubation on a rotary shaker (16 h, 37°C) cultures were extracted with 1 volume ethylacetate, dried and dissolved in 500 µL acetonitrile followed by derivatization with *N*-methyl-*N*-trimethylsilyl-trifluoroacetamid (MSTFA) (55°C, 1 h) and assay products were analyzed by GC-MS. For *in vitro* assays with ArcB, the tag-less purified protein was buffered in 50 mM Tris (pH 7.4). 100 µL assays (2 µg ArcB, 1 mM chorismic acid (Sigma-Aldrich), 100 mM Tris pH 7.4) were incubated at 37°C and stopped after 10 or 60 minutes by incubation at 100°C for 5 min. Stopped assays were adjusted to pH 4 and extracted 2× with 1 volume of ethylacetate. Dried organic phases were dissolved in 40 µL acetonitrile, derivatized with MSTFA (55°C, 1 h) and analyzed by GC-MS. Assay products were detected on a 7890A gas chromatograph (Agilent) equipped with a DB5ht column (30 m×250 µm×0.1 µM, Agilent) coupled with a 5975C mass selective detector (Agilent). The method parameters were the following: Carrier gas: Helium (1 mL/min); injection volume: 1 µL; inlet temperature: 250°C; injection mode splitless; oven starting temperature 5 min 70°C, then 5°C/min to 300°C. Ionization of the analyte molecules were carried out by electron impact ionization at 70 eV. Products were identified with the “Automated Mass Deconvolution and Identification Software” (AMDIS) version 2.64 in combination with the NIST-library.

### ArcT *in vitro* assays

To test the adenylation activity of ArcT *in vitro*, purified tag-less protein was buffered in 20 mM Tris (pH 7.4) and incubated with labeled [γ-^18^O_4_]-ATP, various benzoic acid derivatives (listed in Figure S5), MgCl_2_ and pyrophosphate as described previously [26]. Assays were analyzed by MALDI-MS as reported above.

## Results

### Gene clusters for xanthomonadin and DAR biosynthesis

During our investigations of the DAR biosynthesis we could show that heterologous overexpressed DAR-biosynthesis proteins of *Azoarcus* sp. BH72 led to DAR **7** production in *Escherichia coli* [1], but the corresponding DAR was not detectable in *Azoarcus* extracts. However, *Azoarcus* sp. BH72 extracts contained a yellow hydrophobic compound that showed no color shift with alkali, indicating that the pigment was not a flexirubin [30]. The gene cluster for xanthomonadin biosynthesis (Figure 2.B, Table S4) is conserved within the gammaproteobacteria in several strains of *Xanthomonas* [11]–[13] and was also studied in *L. enzymogenes* [14]. We identified two gene clusters in the genome of the betaproteobacterium *Azoarcus* sp. BH72 (*Rhodocyclales*) encoding proteins with high similarity to those of the xanthomonadin biosynthesis (Figure 2.A, Table 1). Both putative *arc* loci (*azo3910-azo3921* and *azo0253-0264*) were not physically linked. In addition to their already reported DAR biosynthesis gene clusters [1], the putative polyene biosynthesis gene clusters were also found in other betaproteobacteria like *Dechloromonas aromatica* RCB (*Rhodocyclales*) (Figure 2.C, Table S1), *Variovorax paradoxus* S110 (*Burkholderiales*) (Figure 2.D, Table S2) and *Sideroxydans lithotrophicus* ES-1 (*Gallionellales*) (Figure 2.E, Table S3). Within the identified gene clusters several genes are highly conserved (for example a gene encoding a glycosyl transferase, an outer membrane lipoprotein carrier protein (LolA), a phospholipid/glycerol acyltransferase and an adjacent exporter like protein) and we also found these genes in flexirubin biosynthesis gene clusters [31]. BLAST-P and String analysis with proteins from the gene clusters identified in *Azoarcus* revealed that gene clusters encoding these polyene biosynthesis associated genes can be found within several different proteobacteria, bacteria from the *Bacteroidetes* phylum and in one strain from the *Spirochaetes* (Table S7). In addition, 16 of the 39 reported bacterial genomes in Table S7 contained DAR biosynthesis gene clusters, with nine examples where the DAR biosynthesis genes are located within or adjacent to the polyene biosynthesis associated gene cluster.

**Table 1:** Predicted gene clusters for arcuflavin biosynthesis in *Azoarcus* sp. BH72. Domain guided annotation is based on conserved domains detected by BLAST-P.

Gene	Genelocus [azo]	NCBI annotation	domain guided annotation
*arcL*	3910	hypothetical protein	peptidoglycan-binding protein
*arcK*	3911	3-oxoacyl-ACP synthase	ketosynthase
*arcJ*	3912	hypothetical protein	N-terminal beta-ketoacyl synthase domain
*arcI*	3913	hypothetical protein	dehydratase
*arcH*	3914	3-ketoacyl-ACP reductase	reductase
*arcG*	3915	putative Na/H(+) antiporter	Na^+^/H^+^-Antiporter
*arcF*	3916	N-acetyl-gamma-glutamyl-phosphate reductase	N-Acetyl-γ-Glutamyl-Ph-Reduktase
*arcE*	3917	glycosyl transferase	glycosyl transferase
*arcD*	3918	putative phospholipid biosynthesis acyltransferase	acyltransferase
*arcC*	3919	MltA-interacting protein	outer membrane protein
*arcB*	3920	hypothetical protein	chorismatase/XanB2
*arcA*	3921	hypothetical protein	halogenase
*arcM*	0253	hypothetical protein	methyltransferase
*arcN*	0254	polysaccharide deacetylase	PS-deacetylase
*arcO*	0255	hypothetical protein	
*arcP*	0256	hypothetical protein	exporter
*arcQ*	0257	hypothetical protein	lolA
*arcR*	0258	acetyltransferase	acyltransferase
*arcS*	0259	hypothetical protein	dehydratase
*arcT*	0260	hypothetical protein	Acyl-CoA synthetase/AMP- ligases
*arcU*	0261	hypothetical protein	
*arcV*	0262	hypothetical protein	ACP
*arcW*	0263	two-component system histidine kinase	histidinkinase
*arcX*	0264	transcriptional regulator	response regulator
*darK*	0283	hypothetical protein	
*darJ*	0284	putative Beta-ketoacyl synthase family protein	ketosynthase
*darC*	0285	acyl carrier protein	ACP
*darI*	0286	putative ABC transporter permease	ABC-Transporter; Permease
*darH*	0287	hypothetical protein	PilT-Domain
*darG*	0288	hypothetical protein	Prevent host death-protein
*darF*	0289	ABC transporter ATP-binding protein	ABC-Transporter; ATP-Binding.
*darE*	0290	hypothetical protein	BtrH-like peptidase
*darD*	0291	hypothetical protein	conserved hypothetical protein
*darB*	0292	3-oxoacyl-ACP synthase	DAR-cyclase
*darA*	0293	dialkylrecorsinol condensing protein	DAR-aromatase

### Verification of assigned pigment biosynthesis gene clusters

**Figure 3:**
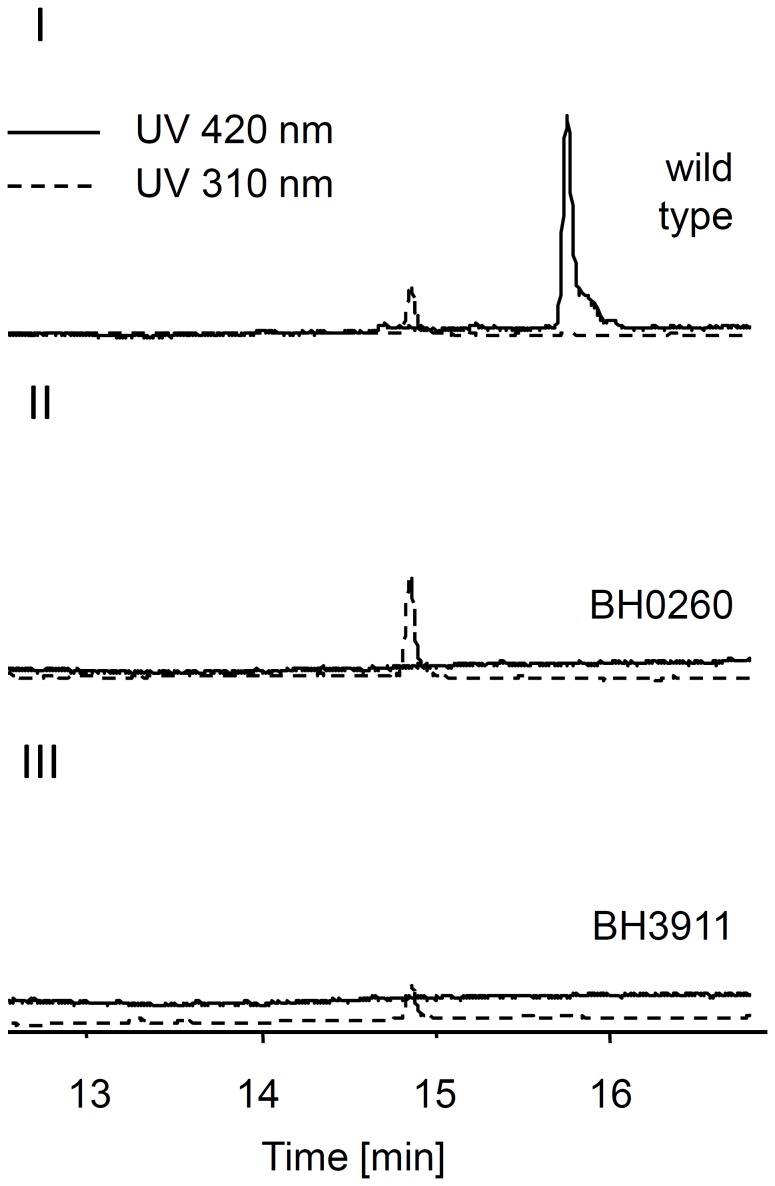
HPLC analysis of *Azoarcus* sp. BH72 wild type and mutant strains. HPLC-UV analysis of wildtype (I), BH0260 (II) and BH3911 extracts with traces showing absorption at 420 nm (solid line) or 310 nm (dotted line). All chromatograms were drawn to the same scale.

Detailed studies with *Xanthomonas* strains showed that the biosynthesis of xanthomonadin depends on the reported gene cluster [11]–[13] (Figure 2.B), which was also shown for the biosynthesis of the xanthomonadin-like pigment from *L. enzymogenes* [14]. Thus we tested whether these gene clusters encoding such homologues are essential for the pigment biosynthesis by insertion of plasmids into gene *arcK* (*azo3911*) or *arcT (azo0260)*, respectively. The resulting mutant strains BH3911 and BH0260 appeared colorless on agar plate (Figure S1), and subsequent acetone extraction and HLPC-UV analysis showed the loss of a UV-signal at 15.7 min in the extracts from the mutants (Figure 3).

### Structure elucidation of the pigment

**Figure 4:**
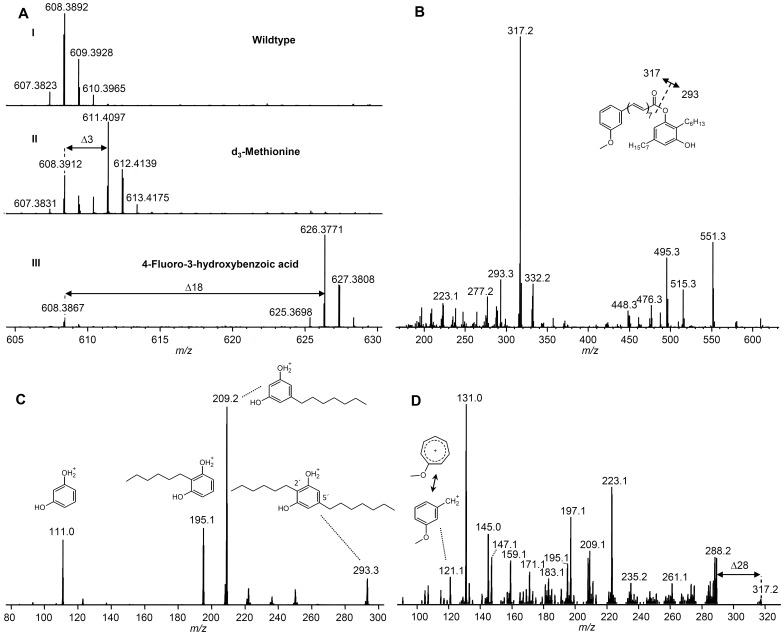
Mass spectra of 8 from *Azoarcus* sp. BH72. **A**: MALDI-orbitrap mass spectra of wildtype **8** (I) and from labeling experiments with d_3_-methionine (II) and 4-Fluoro-3-hydroxybenzoic acid (4F-3HBA) (III). **B**: MALDI-iontrap-MS^2^ of **8**. MALDI-iontrap-MS^3^ of *m/z* 293.3 (**C**) and *m/z* 317.2 (**D**) with *m/z* 608.4 [M]^+•^ as precursor.

Further analysis of acetone extracts of *Azoarcus* sp. BH72 led to the identification of pigment **8** with a mass of *m/z* 608.38633 [M]^+•^ (Figure 4) leading to a sum formula of C_41_H_52_O_4_ (calc. *m/z* 608.38601 [M]^+•^; Δppm 0.5) and UV absorption at 420 nm (Figure S2.A). MALDI-MS^2^ of **8** led to a base peak of *m/z* 317.2 and *m/z* 293.3 (Figure 4.B) and MALDI-MS^3^ of *m/z* 293.3 (Figure 4.C) revealed a fragmentation pattern identical to what has been observed for the heterologous produced DAR **7** [1]. MALDI-MS^3^ of *m/z* 317.2 (Figure 4.D) led to a complex fragmentation pattern with a *m/z* 121 key-fragment, a mass shift of Δ28 Da (-CO) from *m/z* 317.2 and Δ78 Da (-C_6_H_6_) or Δ26 Da (-C_2_H_2_) mass shifts. The conserved structural feature of xanthomonadin is the ω-(3-hydroxyphenyl)-polyene carboxylic acid that is usually methylated at the hydroxyl group. When *Azoarcus* was grown in the presence of L-[*methyl*-^2^H_3_]methionine or 4-fluoro-3-hydroxybenzoic acid (4F-3HBA) (a fluorinated derivative of the putative xanthomonadin precursor 3-hydroxybenzoic acid (3HBA)), an additional M+3 (Figure 4.AII) or M+18 signal (Figure 4.AIII; Figure S2.AII) was detectable, respectively. In contrast, if 3-fluoro-4-hydroxybenzoic acid (3F-4HBA) was fed to the culture no M+18 signal was detected (data not shown). MALDI-MS^2^ analysis of both labeled pigments led to the corresponding Δ3 Da and Δ18 Da mass shifts at the previously *m/z* 317.2 fragments (Figure S2.B) but not at the DAR fragment *m/z* 293.3. Subsequent MS^3^-analysis of the labeled fragments revealed the mass-shifts of the *m/z* 121 key-fragment, which is originating from the polyene moiety (Figure S2.C).

### Initiation of the arcuflavin polyene biosynthesis

**Figure 5:**
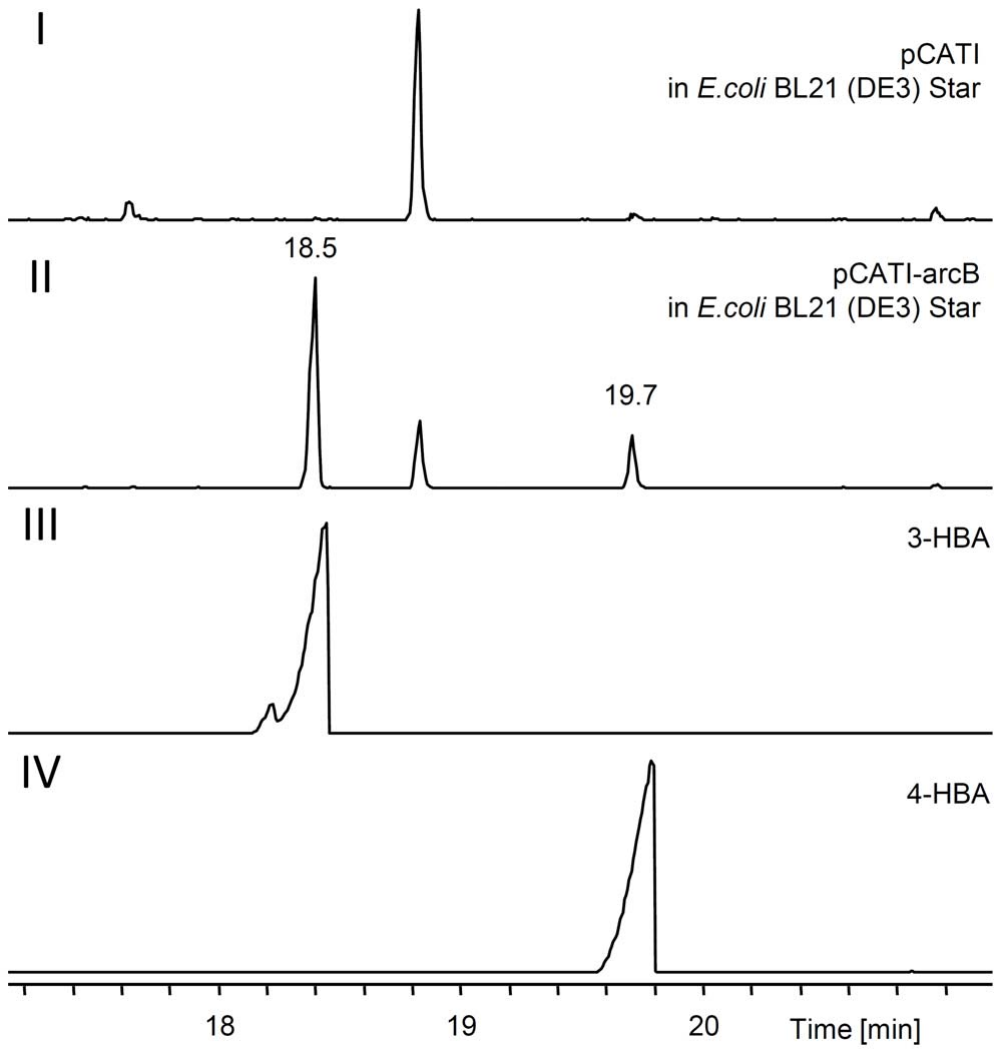
GC-MS analysis of *E. coli* cultures overexpressing *arcB*. Total ion chromatograms of extracted supernatants of *E. coli* BL21 (DE3) Star harboring the vector without insert (I), the *arcB* overexpression plasmid (II) and of 3-hydroxybenzoic acid (3-HBA) (III) and 4-hydroxybenzoic acid (4-HBA) standards (IV).

Previously it was shown that *xanB2* (*Xcc4014*) from *X. campestris* is responsible for production of the diffusible factor (DF) 3-hydroxybenzoic acid (3-HBA), which is essential for xanthomonadin biosynthesis [12], [15]. Recent research revealed that *xanB2* encodes a structurally novel and bifunctional chorismatase converting chorismic acid into 3-HBA, the probable biosynthetic precursor of the xanthomonadin ring moiety, and 4-hydroxybenzoic acid (4-HBA) which is needed for coenzyme Q8 biosynthesis [16], [17]. A homologue of XanB2 in *Azoarcus* is encoded by *arcB* within the postulated pigment biosynthesis gene cluster (Figure 2.A). Like XanB2, ArcB contains a YjgF-like C-terminal domain, which is also present in the chorismate utilizing enzymes Hyg5, FkbO and RapK from *Streptomyces* [32]. Based on their reaction products, these enzymes were sorted in three distinct types of chorismatases [33]. The FkbO type produces 3,4-dihydroxycyclohexa-1,5-dienoic acid, the Hyg5-type forms 3-HBA whereas the XanB2-type converts chorismate to 3-HBA and 4-HBA [33]. Since from sequence alignment alone it is actually not possible to predict accurately the reaction catalyzed by these chorismate-converting enzymes and to get experimental evidence for the catalyzed reaction, *arcB* was overexpressed in *E. coli* and the culture supernatants were extracted. GC-MS analysis of these extracts (Figure 5) revealed the formation of compounds with similar retention times and mass spectra (Figure S3.A) as 3-HBA and 4-HBA standards. Subsequent purification of ArcB (Figure S4) allowed its incubation with chorismic acid. In all assays 4-HBA was formed non-enzymatically (Figure S3.BIV) as reported before [16], [34], whereas 3-HBA was only detectable in assays containing ArcB (Figure S3.III). Since it was postulated that xanthomonadin biosynthesis is performed by a type II fatty acid synthase like biosynthesis using 3-HBA as polyene precursor [15], 3-HBA should be activated for the polyketide synthase (PKS) machinery. Such a reaction might be performed by the putative AMP-ligase encoded by *Xcc4015* in *X. campestris* pv. *campestris*, which is essential for the pigment biosynthesis in this organism [15]. The homologue ArcT, encoded in the pigment gene cluster from *Azoarcus* sp. BH72, was overexpressed in *E. coli* and purified (Figure S4). The adenylation activity of ArcT was tested with a method measuring the isotopic back exchange of unlabelled pyrophosphate into [γ-^18^O_4_]-ATP if the enzyme adenylates a substrate [26]. MALDI-MS analysis showed ATP-label exchange in assays with 3-HBA and various other benzoic acids with modifications in 4-position which was not detected if no substrate, 3-methoxybenzoic acid or 2-substituted benzoic acids were added (Figure S5).

## Discussion

In previous studies the xanthomonadin biosynthesis gene cluster was found in genomes of other gamma- and betaproteobacteria and homologues of the chorismate-utilizing enzyme XanB2 (Xcc4014) were also found in genomes outside of the proteobacteria [16]. However, only the gene cluster of the closely related bacterium *L. enzymogenes* (family *xanthomonadaceae*) was studied and a non-brominated pigment was found in this bacterium, which is most likely xanthomonadin-like [14]. Here we identified for the first time similar polyene biosynthesis gene clusters in the genome of *Azoarcus* sp. BH72 which additionally contains a DAR-biosynthesis gene cluster. Our results with plasmid insertion mutants show that both xanthomonadin-like biosynthesis gene clusters, although they are not physically linked, are essential for pigment **8** biosynthesis in *Azoarcus* sp. BH72, indicating that its product is indeed a xanthomonadin-like compound.

Results from MS^2^ experiments with pigment **8** show that its fragmentation is similar to those of flexirubin where the basepeak was shown to result from the cleavage of the polyene-DAR ester bond [31]. Therefore we predict that DAR **7** and the xanthomonadin-like polyene are connected in a flexirubin-like manner by an ester bond between the polyene carboxylic acid and a phenolic hydroxyl group. Similar to flexirubin, the formation of a radical cation was observed that might be explained by direct absorption of the laser light by the polyene of pigment **8** instead of matrix mediated ionization mechanisms. Since the MS^3^ of the assigned DAR-fragment *m/z* 293.3 is identical to those from heterologous produced DAR **7** [1], we conclude that the *Azoarcus* pigment **8** has a DAR moiety identical to **7**. It was postulated that 3-HBA is a biosynthetic intermediate of the xanthomonadin biosynthesis [15]. Our labeling result with 4F-3HBA now confirms that this is indeed the case in the biosynthesis of pigment **8** from *Azoarcus* which additionally contains a methionine derived methylgroup. Furthermore the MS^3^ of the assigned polyene fragment *m/z 317.2* shows mass shifts of Δ28 Da (CO), Δ78 Da (C_6_H_6_) or Δ26 Da (C_2_H_2_) (Figure S2.C), already known from mass spectrometric fragmentation of flexirubin-like polyenes [31], [35]. These results suggest the structure of a nonbrominated aryl-heptaene for the polyene moiety of **8**, reflecting xanthomonadin group-11 previously found in *Xanthomonas* [36], [37]. Based on these findings we conclude that a new xanthomonadin-DAR hybrid has been characterized, which we named arcuflavin (from *Azoarcus* and latin for yellow) and propose it to have the structure of **8**. Since arcuflavin is only produced in low amounts and becomes insoluble in various organic solvents after enrichment, similar to the *L. enzymogenes* xanthomonadin-like pigment [14], further structure elucidation by means of NMR was not possible.

Combinations of xanthomonadin-like polyene gene cluster and DAR-gene cluster were also found in *D. aromatica* RCB, three strains of *V. paradoxus* and *S. lithotrophicus* ES-1. Whereas in previous genome screenings only the xanthomonadin-like gene clusters from *D. aromatica* RCB and *V. paradoxus* EPS and S110 were found [14], [16], we could now identify their DAR gene clusters and it will be interesting to see in future studies if the combination of both gene cluster types leads to the identification of polyene-DAR hybrids also in these organisms. In addition we found that genes associated with known polyene biosynthesis gene clusters with and without DAR biosynthesis encoding genes can be found within a wide range of bacterial genomes including medically important taxa like *Burkholderia*, *Vibrio*, *Escherichia* and *Pseudomonas* (Table S7). We therefore predict that more pigments like flexirubin, xanthomonadin or arcuflavin will be found in these organisms and that these compounds might also be involved in the pathophysiology of these organisms. Moreover, our findings suggest that these pigments cannot be used as chemotaxonomic markers, especially in the case of flexirubin for the *Bacteroidetes* phylum, as they seem to be much more widespread than originally thought.

We investigated the first steps of the arcuflavin-polyene biosynthesis and could show that ArcB has *in vitro* the assumed catalytic activity of a chorismate-utilizing enzyme that produces 3-HBA and 4-HBA. This is similar to the reaction performed by its homologue in the xanthomonadin biosynthesis pathway [16], [17] and makes it the third biochemically characterized example of the XanB2-type of chorismate-utilizing enzymes. Furthermore the activity of the AMP-ligase ArcT with 3-HBA could be shown *in vitro*. The result that 3-methoxybenzoic acid was not a good substrate for ArcT suggests that the methylation of pigment **8** takes place after the activation of the 3-HBA starter. Although ArcT showed *in vitro* a low substrate specificity, no other derivatives of arcuflavin **8** were found in the extracts of *Azoarcus*, showing that *in vivo* the arcuflavin-biosynthesis machinery is more selective in the precursor or intermediate selection.

## Conclusions

Due to their structural similarity one might conclude a functional similarity for arcuflavin with xanthomonadins and DARs. Xanthomonadins from *Xanthomonas oryzae pv. oryzae* and *X. campestris pv. campestris* are located in the bacterial outer membrane [13], [38]and have been shown to protect their producers from photooxidative damage and lipid peroxidation [18]–[20]. On the other hand, the DARs DB-2073 (**1**) and resorstatin (**2**) were reported to act as free radical scavengers to protect against lipid peroxidation [4] and show striking structural similarity to the DAR from arcuflavin. Therefore DAR or polyene-DAR hybrids like arcuflavin might protect the cell against oxidative damage, suggesting the fusion of two protective moieties in polyene-DAR hybrids. Bacteria from the genus *Xanthomonas* are well known plant pathogens, *Azoarcus* sp. BH72 is a diazotroph endophyte of rice and other grasses [39], whereas *V. paradoxus* S110 is a growth-promoting endophyte of various plants [40]. As transient production of reactive oxygen species is found in most plant-microorganism interactions [41], arcuflavin and similar polyene pigments might play a role in establishing or maintaining the symbiotic life of the bacterium with its host.

## Supporting Information

Table S1 **Predicted gene clusters for arcuflavine-like biosynthesis in** ***Dechloromonas aromatica*** **RCB.**
(DOCX)

Table S2 **Predicted gene clusters for arcuflavin-like biosynthesis in** ***Variovorax paradoxus*** **S110.**
(DOCX)

Table S3 **Predicted gene clusters for arcuflavin-like biosynthesis in** ***Sideroxydans lithotrophicus*** **ES-1.**
(DOCX)

Table S4 **Gene cluster for xanthomonadin biosynthesis in** ***Xanthomonas campestris*** **pv.** ***campestris*** **ATCC33913.**
(DOCX)

Table S5 **Strains and plasmids used in this work.**
(DOCX)

Table S6 **Primers and PCR products used in this study.**
(DOCX)

Table S7 **Predicted gene clusters encoding polyene-associated biosynthesis and DAR-biosynthesis proteins.**
(DOCX)

Figure S1 **Phenotype of** ***Azoarcus*** **sp. BH72 wildtype and mutant strains.** Growth and pigmentation of wildtype *Azoarcus* sp. BH72 and insertional mutants on VM-agar (with ethanol) plate (left) or a kanamycin containing VM-agar (with ethanol) plate (right).
(TIF)

Figure S2 **HPLC-MS-analysis and mass spectra of 8 from** ***Azoarcus*** **sp. BH72.** **A**: HPLC-MS-analysis of wildtype **8** (I) and after feeding with 4-fluoro-3-hydroxybenzoic acid (4F-3HBA) (II) in raw extracts. Traces show EIC of *m/z* 607.5 [M-H^+^] (orange), *m/z* 625.5 [M-H^+^] (green) and UV at 420 nm (blue). Chromatograms are drawn to the same scale. **B**: MALDI-iontrap-MS^2^ of wildtype **8** (I), *m/z* 611.4 [M]^+•^ (II) and *m/z* 626.4 [M]^+•^ (III) from feeding experiments with d_3_-methionine or 4F-3HBA, respectively. **C**: MALDI-iontrap-MS^3^ mass spectra of the polyene-fragments *m/z* 317.3 (I), *m/z* 320.2 (II) and *m/z* 335.3 (III), that were obtained from the precursors *m/z* 608.4 [M]^+•^(I), *m/z* 611.4 [M]^+•^ (II) and *m/z* 626.4 [M]^+•^(III). Mass shifts mentioned in the text are indicated.
(TIF)

Figure S3 **GC-MS analysis of** ***E. coli*** **cultures overexpressing** ***arcB*** **and** ***in vitro*** **assays with purified ArcB.** **A**: Mass spectra of 3-hydroxybenzoic acid (3-HBA) standard (I) and the compounds identified at min 18.5 (II) and min 19.7 (III) in *E. coli* cultures overexpressing *arcB*. **B**: Total ion chromatograms of 4-hydroxybenzoic acid (4-HBA) standard (I) and 3-HBA standard (II) and chorismic acid containing *in vitro* assays incubated with (III) or without (IV) ArcB and stopped after 10 (blue) or 60 min (red) incubation, respectively.
(TIF)

Figure S4 **SDS-PAGE analysis of purified** ***Azoarcus*** **proteins.** **M**: PageRuler™ Unstained protein ladder (Fermentas). **Lane 1–8**: Fractions of ArcB purification with the purified protein in Lane 8 (expected size 35,24 kDA) **Lane 10**: Purified ArcT (expected size: 47,1 kDa). Stars indicate protein bands with the expected size.
(TIF)

Figure S5 **MALDI-MS analysis of [γ-^18^O_4_]-ATP containing assays with ArcT and various substrates.** A mass shift from *m/z* 514 to *m/z* 506 indicates the exchange of the ATP-label and therefore activity of ArcT with the substrate. Substrates: **A**: none **B**: 3-hydroxybenzoic acid **C**: 4-fluoro-3-hydroxybenzoic acid **D**: 4-fluorobenzoic acid **E**: 4-hydroxybenzoic acid **F**: 4-methoxybenzoic acid **G**: 3-methoxybenzoic acid **H**: 2-methoxybenzoic acid **I**: 2,3-dihydroxybenzoic acid **J**: 3,5- dihydroxybenzoic acid **K**: benzoic acid **L**: *p*-coumaric acid **M**: *E*-cinnamic acid. Colours indicate if no (red), <50% (orange) or >50% label exchange (green) was detected.
(TIF)
