# Supplementary material for: Identification and Biosynthesis of a Novel Xanthomonadin-Dialkylresorcinol-Hybrid from Azoarcus sp. BH72
Source: PLoS One. 2014 Mar 11;9(3):e90922. doi: 10.1371/journal.pone.0090922 (PMC3949708; doi:10.1371/journal.pone.0090922)
Supplement: Table S1 — Predicted gene clusters for arcuflavine-like biosynthesis in Dechloromonas aromatica RCB. (DOCX) [file pone.0090922.s001.docx]

| **Gene locus [daro_]** | **NCBI annotation** | **domain guided annotation** |
| --- | --- | --- |
| 4199 | cobalamin B12-binding:radical SAM family protein | methyltransferase |
| 4198 | hypothetical protein |  |
| 4197 | monooxygenase, FAD-binding:FAD dependent oxidoreductase:tryptophan halogenase | halogenase/reductase |
| 4196 | endoribonuclease L-PSP | chorismatase |
| 4195 | outer membrane protein | MltA-interacting protein |
| 4194 | phospholipid/glycerol acyltransferase | acyltransferase |
| 4193 | glycosyl transferase | glycosyl transferase |
| 4192 | CBS:HPP |  |
| 4191 | Sodium/hydrogen exchanger | Na^+^/H^+^-Antiporter |
| 4190 | thioredoxin |  |
| 4189 | hypothetical protein |  |
| 4188 | 3-ketoacyl-ACP reductase | reductase |
| 4187 | hypothetical protein | FabA like dehydratase |
| 4186 | hypothetical protein | N-terminal beta-ketoacyl synthase domain |
| 4185 | 3-oxoacyl-ACP synthase | ketosynthase |
| 4184 | extracellular ligand-binding receptor |  |
| 4183 | hydratase/decarboxylase |  |
| 4182 | anaerobic C4-dicarboxylate transporter |  |
| 4181 | hypothetical protein |  |
| 4180 | hypothetical protein | methyltransferase |
| 4179 | polysaccharide deacetylase |  |
| 4178 | hypothetical protein |  |
| 4177 | transmembrane protein | exporter |
| 4176 | hypothetical protein |  |
| 4175 | lauroyl/myristoyl acyltransferase involved in LPS biosynthesis | phospholipid/glycerol acyltransferase |
| 4174 | hypothetical protein | dehydratase |
| 4173 | AMP-dependent synthetase/ligase | Acyl-CoA synthetase/AMP- ligases |
| 4172 | transmembrane protein |  |
| 4171 | acyl carrier protein | ACP |
| 4170 | 3-oxoacyl-ACP synthase | ketosynthase |
| 4169 | acyl carrier protein | ACP |
| 4168 | phosphoesterase, PA-phosphatase related |  |
| 2375 | hypothetical protein |  |
| 2374 | Beta-ketoacyl synthase | ketosynthase |
| 2373 | acyl carrier protein | ACP |
| 2372 | hypothetical protein | ABC-Transporter; Permease |
| 2371 | ABC transporter related | ABC-Transporter; ATP-Bindingg. |
| 2370 | hypothetical protein | BtrH-like peptidase |
| 2369 | hypothetical protein | conserved hypothetical protein |
| 2368 | 3-oxoacyl-ACP synthase | DAR-cyclase |
| 2367 | hypothetical protein | DAR-aromatase |

Table S1
