# Supplementary material for: Identification and Biosynthesis of a Novel Xanthomonadin-Dialkylresorcinol-Hybrid from Azoarcus sp. BH72
Source: PLoS One. 2014 Mar 11;9(3):e90922. doi: 10.1371/journal.pone.0090922 (PMC3949708; doi:10.1371/journal.pone.0090922)
Supplement: Table S2 — Predicted gene clusters for arcuflavin-like biosynthesis in Variovorax paradoxus S110. (DOCX) [file pone.0090922.s002.docx]

| **Genelocus [vapar_]** | **NCBI annotation** | **domain guided annotation** |
| --- | --- | --- |
| 0260 | FAD-binding monooxygenase |  |
| 0261 | hypothetical protein | N-terminal beta-ketoacyl synthase domain |
| 0262 | 3-oxoacyl-(acyl carrier protein) synthase I | ketosynthase |
| 0263 | major facilitator superfamily protein | muropeptide transporter |
| 0264 | peptidase M48 Ste24p | peptidase |
| 2682 | type 11 methyltransferase | UbiE-like methyltransferase |
| 2681 | endoribonuclease L-PSP | chorismatase |
| 2680 | phospholipid/glycerol acyltransferase | acyltransferase |
| 2679 | family 2 glycosyl transferase | glycosyl transferase |
| 2678 | 3-ketoacyl-(acyl-carrier-protein) reductase | reductase |
| 2677 | potential 3-hydroxydecyl-(acyl carrier protein) dehydratase | dehydratase |
| 2676 | type 11 methyltransferase | methyltransferase |
| 2675 | polysaccharide deacetylase |  |
| 2674 | hypothetical protein | exporter |
| 2673 | hypothetical protein |  |
| 2672 | hypothetical protein | phospholipid/glycerol acyltransferase |
| 2671 | beta-hydroxyacyl-(acyl-carrier-protein)  dehydratase FabA/FabZ | Acyl-CoA synthetase/AMP- ligases |
| 2670 | transmembrane protein |  |
| 2669 | acyl carrier protein | ACP |
| 3382 | hypothetical protein |  |
| 3383 | beta-ketoacyl synthase | ketosynthase |
| 3384 | phosphopantetheine-binding | ACP |
| 3385 | ABC transporter | ABC-Transporter; Permease |
| 3386 | ABC transporter | ABC-Transporter; ATP-Bindingg. |
| 3387 | hypothetical protein | BtrH-like peptidase |
| 3388 | hypothetical protein | conserved hypothetical protein |
| 3389 | 3-oxoacyl-(acyl carrier protein) synthase III | DAR-cyclase |
| 3390 | hypothetical protein | DAR-aromatase |

Table S2
