# Supplementary material for: Identification and Biosynthesis of a Novel Xanthomonadin-Dialkylresorcinol-Hybrid from Azoarcus sp. BH72
Source: PLoS One. 2014 Mar 11;9(3):e90922. doi: 10.1371/journal.pone.0090922 (PMC3949708; doi:10.1371/journal.pone.0090922)
Supplement: Table S3 — Predicted gene clusters for arcuflavin-like biosynthesis in Sideroxydans lithotrophicus ES-1. (DOCX) [file pone.0090922.s003.docx]

| **Genelocus [Slit_]** | **NCBI annotation** | **domain guided annotation** |
| --- | --- | --- |
| 349 | radical SAM domain protein | methyltransferase |
| 350 | beta-ketoacyl synthase | ketosynthase |
| 351 | hypothetical protein | N-terminal beta-ketoacyl synthase domain |
| 352 | hypothetical protein | hot-dog fold dehydratase (FabA) or 4-hydroxybenzoyl-CoA thioesterase |
| 353 | short-chain dehydrogenase/reductase SDR | reductase |
| 354 | hypothetical protein | ACP |
| 355 | ACP | ACP |
| 356 | beta-ketoacyl synthase | ketosynthase |
| 357 | hypothetical protein | benzoate oxygenase |
| 358 | hypothetical protein | DAR-aromatase DarA |
| 359 | 3-oxoacyl-ACP synthase III | DAR cyclase DarB |
| 360 | hypothetical protein | conserved hypothetical protein |
| 361 | hypothetical protein | BtrH-like peptidase |
| 362 | methyltransferase type 11 | methyltransferase |
| 363 | hypothetical protein |  |
| 364 | AMP-dependent synthetase and ligase | Acyl-CoA synthetase/AMP- ligases |
| 365 | hypothetical protein | hot-dog fold dehydratase (FabA) or 4-hydroxybenzoyl-CoA thioesterase |
| 366 | lipid A biosynthesis acyltransferase |  |
| 367 | transmembrane protein |  |
| 368 | hypothetical protein | exporter |
| 369 | hypothetical protein | SmpA / OmlA family |
| 370 | hypothetical protein |  |
| 371 | hypothetical protein |  |
| 372 | hypothetical protein | chorismatase |
| 373 | MltA-interacting MipA family protein |  |
| 374 | phospholipid/glycerol acyltransferase | acyltransferase |
| 375 | glycosyl transferase family 2 | glycosyl transferase |
| 376 | radical SAM domain protein | methyltransferase |

Table S3
