# Supplementary material for: Identification and Biosynthesis of a Novel Xanthomonadin-Dialkylresorcinol-Hybrid from Azoarcus sp. BH72
Source: PLoS One. 2014 Mar 11;9(3):e90922. doi: 10.1371/journal.pone.0090922 (PMC3949708; doi:10.1371/journal.pone.0090922)
Supplement: Table S4 — Gene cluster for xanthomonadin biosynthesis in Xanthomonas campestris pv. campestris ATCC33913. (DOCX) [file pone.0090922.s004.docx]

Table S4

| ***X. campestris* pv. *campestris* ATCC33913** | | | | ***Azoarcus* sp. BH72** | | | | | | | ***D. aromatica* RCB** | | | | | | | | ***V. paradoxus* S110** | | | | | | | | | ***S. lithotrophicus* ES-1** | | | | | | |
| --- | --- | --- | --- | --- | --- | --- | --- | --- | --- | --- | --- | --- | --- | --- | --- | --- | --- | --- | --- | --- | --- | --- | --- | --- | --- | --- | --- | --- | --- | --- | --- | --- | --- | --- |
| Genelocus [Xcc_] | NCBI annotation | domain guided annotation | Accession number | Gene [Arc#] | [Max score](http://blast.ncbi.nlm.nih.gov/Blast.cgi?CMD=Get&ALIGNMENTS=0&ALIGNMENT_VIEW=Pairwise&CDD_SEARCH_STATE=4&DATABASE_SORT=0&DESCRIPTIONS=100&DYNAMIC_FORMAT=on&ENTREZ_QUERY=txid471854%20%5bORGN%5d%20OR%20txid62928%20%5bORGN%5d%20OR%20txid340%20%5bORGN%5d%20OR%20txid485918%20%5bORGN%5d&FIRST_QUERY_NUM=0&FORMAT_OBJECT=Alignment&FORMAT_PAGE_TARGET=&FORMAT_TYPE=HTML&GET_SEQUENCE=yes&I_THRESH=&MASK_CHAR=2&MASK_COLOR=1&NEW_VIEW=yes&NUM_OVERVIEW=100&OLD_BLAST=false&PAGE=Proteins&QUERY_INDEX=0&QUERY_NUMBER=0&RESULTS_PAGE_TARGET=&RID=GRGSF37701R&SHOW_LINKOUT=yes&SHOW_OVERVIEW=yes&STEP_NUMBER=&WORD_SIZE=3&OLD_VIEW=false&DISPLAY_SORT=1&HSP_SORT=1) | [Total score](http://blast.ncbi.nlm.nih.gov/Blast.cgi?CMD=Get&ALIGNMENTS=0&ALIGNMENT_VIEW=Pairwise&CDD_SEARCH_STATE=4&DATABASE_SORT=0&DESCRIPTIONS=100&DYNAMIC_FORMAT=on&ENTREZ_QUERY=txid471854%20%5bORGN%5d%20OR%20txid62928%20%5bORGN%5d%20OR%20txid340%20%5bORGN%5d%20OR%20txid485918%20%5bORGN%5d&FIRST_QUERY_NUM=0&FORMAT_OBJECT=Alignment&FORMAT_PAGE_TARGET=&FORMAT_TYPE=HTML&GET_SEQUENCE=yes&I_THRESH=&MASK_CHAR=2&MASK_COLOR=1&NEW_VIEW=yes&NUM_OVERVIEW=100&OLD_BLAST=false&PAGE=Proteins&QUERY_INDEX=0&QUERY_NUMBER=0&RESULTS_PAGE_TARGET=&RID=GRGSF37701R&SHOW_LINKOUT=yes&SHOW_OVERVIEW=yes&STEP_NUMBER=&WORD_SIZE=3&OLD_VIEW=false&DISPLAY_SORT=2&HSP_SORT=1) | [coverage [%]](http://blast.ncbi.nlm.nih.gov/Blast.cgi?CMD=Get&ALIGNMENTS=0&ALIGNMENT_VIEW=Pairwise&CDD_SEARCH_STATE=4&DATABASE_SORT=0&DESCRIPTIONS=100&DYNAMIC_FORMAT=on&ENTREZ_QUERY=txid471854%20%5bORGN%5d%20OR%20txid62928%20%5bORGN%5d%20OR%20txid340%20%5bORGN%5d%20OR%20txid485918%20%5bORGN%5d&FIRST_QUERY_NUM=0&FORMAT_OBJECT=Alignment&FORMAT_PAGE_TARGET=&FORMAT_TYPE=HTML&GET_SEQUENCE=yes&I_THRESH=&MASK_CHAR=2&MASK_COLOR=1&NEW_VIEW=yes&NUM_OVERVIEW=100&OLD_BLAST=false&PAGE=Proteins&QUERY_INDEX=0&QUERY_NUMBER=0&RESULTS_PAGE_TARGET=&RID=GRGSF37701R&SHOW_LINKOUT=yes&SHOW_OVERVIEW=yes&STEP_NUMBER=&WORD_SIZE=3&OLD_VIEW=false&DISPLAY_SORT=4&HSP_SORT=0) | [E value](http://blast.ncbi.nlm.nih.gov/Blast.cgi?CMD=Get&ALIGNMENTS=0&ALIGNMENT_VIEW=Pairwise&CDD_SEARCH_STATE=4&DATABASE_SORT=0&DESCRIPTIONS=100&DYNAMIC_FORMAT=on&ENTREZ_QUERY=txid471854%20%5bORGN%5d%20OR%20txid62928%20%5bORGN%5d%20OR%20txid340%20%5bORGN%5d%20OR%20txid485918%20%5bORGN%5d&FIRST_QUERY_NUM=0&FORMAT_OBJECT=Alignment&FORMAT_PAGE_TARGET=&FORMAT_TYPE=HTML&GET_SEQUENCE=yes&I_THRESH=&MASK_CHAR=2&MASK_COLOR=1&NEW_VIEW=yes&NUM_OVERVIEW=100&OLD_BLAST=false&PAGE=Proteins&QUERY_INDEX=0&QUERY_NUMBER=0&RESULTS_PAGE_TARGET=&RID=GRGSF37701R&SHOW_LINKOUT=yes&SHOW_OVERVIEW=yes&STEP_NUMBER=&WORD_SIZE=3&OLD_VIEW=false&DISPLAY_SORT=0&HSP_SORT=0) | [identity [%]](http://blast.ncbi.nlm.nih.gov/Blast.cgi?CMD=Get&ALIGNMENTS=0&ALIGNMENT_VIEW=Pairwise&CDD_SEARCH_STATE=4&DATABASE_SORT=0&DESCRIPTIONS=100&DYNAMIC_FORMAT=on&ENTREZ_QUERY=txid471854%20%5bORGN%5d%20OR%20txid62928%20%5bORGN%5d%20OR%20txid340%20%5bORGN%5d%20OR%20txid485918%20%5bORGN%5d&FIRST_QUERY_NUM=0&FORMAT_OBJECT=Alignment&FORMAT_PAGE_TARGET=&FORMAT_TYPE=HTML&GET_SEQUENCE=yes&I_THRESH=&MASK_CHAR=2&MASK_COLOR=1&NEW_VIEW=yes&NUM_OVERVIEW=100&OLD_BLAST=false&PAGE=Proteins&QUERY_INDEX=0&QUERY_NUMBER=0&RESULTS_PAGE_TARGET=&RID=GRGSF37701R&SHOW_LINKOUT=yes&SHOW_OVERVIEW=yes&STEP_NUMBER=&WORD_SIZE=3&DISPLAY_SORT=3&HSP_SORT=3) | Accession number | | Genelocus [daro_] | [Max score](http://blast.ncbi.nlm.nih.gov/Blast.cgi?CMD=Get&ALIGNMENTS=0&ALIGNMENT_VIEW=Pairwise&CDD_SEARCH_STATE=4&DATABASE_SORT=0&DESCRIPTIONS=100&DYNAMIC_FORMAT=on&ENTREZ_QUERY=txid471854%20%5bORGN%5d%20OR%20txid62928%20%5bORGN%5d%20OR%20txid340%20%5bORGN%5d%20OR%20txid485918%20%5bORGN%5d&FIRST_QUERY_NUM=0&FORMAT_OBJECT=Alignment&FORMAT_PAGE_TARGET=&FORMAT_TYPE=HTML&GET_SEQUENCE=yes&I_THRESH=&MASK_CHAR=2&MASK_COLOR=1&NEW_VIEW=yes&NUM_OVERVIEW=100&OLD_BLAST=false&PAGE=Proteins&QUERY_INDEX=0&QUERY_NUMBER=0&RESULTS_PAGE_TARGET=&RID=GRGSF37701R&SHOW_LINKOUT=yes&SHOW_OVERVIEW=yes&STEP_NUMBER=&WORD_SIZE=3&OLD_VIEW=false&DISPLAY_SORT=1&HSP_SORT=1) | [Total score](http://blast.ncbi.nlm.nih.gov/Blast.cgi?CMD=Get&ALIGNMENTS=0&ALIGNMENT_VIEW=Pairwise&CDD_SEARCH_STATE=4&DATABASE_SORT=0&DESCRIPTIONS=100&DYNAMIC_FORMAT=on&ENTREZ_QUERY=txid471854%20%5bORGN%5d%20OR%20txid62928%20%5bORGN%5d%20OR%20txid340%20%5bORGN%5d%20OR%20txid485918%20%5bORGN%5d&FIRST_QUERY_NUM=0&FORMAT_OBJECT=Alignment&FORMAT_PAGE_TARGET=&FORMAT_TYPE=HTML&GET_SEQUENCE=yes&I_THRESH=&MASK_CHAR=2&MASK_COLOR=1&NEW_VIEW=yes&NUM_OVERVIEW=100&OLD_BLAST=false&PAGE=Proteins&QUERY_INDEX=0&QUERY_NUMBER=0&RESULTS_PAGE_TARGET=&RID=GRGSF37701R&SHOW_LINKOUT=yes&SHOW_OVERVIEW=yes&STEP_NUMBER=&WORD_SIZE=3&OLD_VIEW=false&DISPLAY_SORT=2&HSP_SORT=1) | [coverage [%]](http://blast.ncbi.nlm.nih.gov/Blast.cgi?CMD=Get&ALIGNMENTS=0&ALIGNMENT_VIEW=Pairwise&CDD_SEARCH_STATE=4&DATABASE_SORT=0&DESCRIPTIONS=100&DYNAMIC_FORMAT=on&ENTREZ_QUERY=txid471854%20%5bORGN%5d%20OR%20txid62928%20%5bORGN%5d%20OR%20txid340%20%5bORGN%5d%20OR%20txid485918%20%5bORGN%5d&FIRST_QUERY_NUM=0&FORMAT_OBJECT=Alignment&FORMAT_PAGE_TARGET=&FORMAT_TYPE=HTML&GET_SEQUENCE=yes&I_THRESH=&MASK_CHAR=2&MASK_COLOR=1&NEW_VIEW=yes&NUM_OVERVIEW=100&OLD_BLAST=false&PAGE=Proteins&QUERY_INDEX=0&QUERY_NUMBER=0&RESULTS_PAGE_TARGET=&RID=GRGSF37701R&SHOW_LINKOUT=yes&SHOW_OVERVIEW=yes&STEP_NUMBER=&WORD_SIZE=3&OLD_VIEW=false&DISPLAY_SORT=4&HSP_SORT=0) | [E value](http://blast.ncbi.nlm.nih.gov/Blast.cgi?CMD=Get&ALIGNMENTS=0&ALIGNMENT_VIEW=Pairwise&CDD_SEARCH_STATE=4&DATABASE_SORT=0&DESCRIPTIONS=100&DYNAMIC_FORMAT=on&ENTREZ_QUERY=txid471854%20%5bORGN%5d%20OR%20txid62928%20%5bORGN%5d%20OR%20txid340%20%5bORGN%5d%20OR%20txid485918%20%5bORGN%5d&FIRST_QUERY_NUM=0&FORMAT_OBJECT=Alignment&FORMAT_PAGE_TARGET=&FORMAT_TYPE=HTML&GET_SEQUENCE=yes&I_THRESH=&MASK_CHAR=2&MASK_COLOR=1&NEW_VIEW=yes&NUM_OVERVIEW=100&OLD_BLAST=false&PAGE=Proteins&QUERY_INDEX=0&QUERY_NUMBER=0&RESULTS_PAGE_TARGET=&RID=GRGSF37701R&SHOW_LINKOUT=yes&SHOW_OVERVIEW=yes&STEP_NUMBER=&WORD_SIZE=3&OLD_VIEW=false&DISPLAY_SORT=0&HSP_SORT=0) | [identity [%]](http://blast.ncbi.nlm.nih.gov/Blast.cgi?CMD=Get&ALIGNMENTS=0&ALIGNMENT_VIEW=Pairwise&CDD_SEARCH_STATE=4&DATABASE_SORT=0&DESCRIPTIONS=100&DYNAMIC_FORMAT=on&ENTREZ_QUERY=txid471854%20%5bORGN%5d%20OR%20txid62928%20%5bORGN%5d%20OR%20txid340%20%5bORGN%5d%20OR%20txid485918%20%5bORGN%5d&FIRST_QUERY_NUM=0&FORMAT_OBJECT=Alignment&FORMAT_PAGE_TARGET=&FORMAT_TYPE=HTML&GET_SEQUENCE=yes&I_THRESH=&MASK_CHAR=2&MASK_COLOR=1&NEW_VIEW=yes&NUM_OVERVIEW=100&OLD_BLAST=false&PAGE=Proteins&QUERY_INDEX=0&QUERY_NUMBER=0&RESULTS_PAGE_TARGET=&RID=GRGSF37701R&SHOW_LINKOUT=yes&SHOW_OVERVIEW=yes&STEP_NUMBER=&WORD_SIZE=3&DISPLAY_SORT=3&HSP_SORT=3) | Accession number | | Genelocus [vapar_] | [Max score](http://blast.ncbi.nlm.nih.gov/Blast.cgi?CMD=Get&ALIGNMENTS=0&ALIGNMENT_VIEW=Pairwise&CDD_SEARCH_STATE=4&DATABASE_SORT=0&DESCRIPTIONS=100&DYNAMIC_FORMAT=on&ENTREZ_QUERY=txid471854%20%5bORGN%5d%20OR%20txid62928%20%5bORGN%5d%20OR%20txid340%20%5bORGN%5d%20OR%20txid485918%20%5bORGN%5d&FIRST_QUERY_NUM=0&FORMAT_OBJECT=Alignment&FORMAT_PAGE_TARGET=&FORMAT_TYPE=HTML&GET_SEQUENCE=yes&I_THRESH=&MASK_CHAR=2&MASK_COLOR=1&NEW_VIEW=yes&NUM_OVERVIEW=100&OLD_BLAST=false&PAGE=Proteins&QUERY_INDEX=0&QUERY_NUMBER=0&RESULTS_PAGE_TARGET=&RID=GRGSF37701R&SHOW_LINKOUT=yes&SHOW_OVERVIEW=yes&STEP_NUMBER=&WORD_SIZE=3&OLD_VIEW=false&DISPLAY_SORT=1&HSP_SORT=1) | [Total score](http://blast.ncbi.nlm.nih.gov/Blast.cgi?CMD=Get&ALIGNMENTS=0&ALIGNMENT_VIEW=Pairwise&CDD_SEARCH_STATE=4&DATABASE_SORT=0&DESCRIPTIONS=100&DYNAMIC_FORMAT=on&ENTREZ_QUERY=txid471854%20%5bORGN%5d%20OR%20txid62928%20%5bORGN%5d%20OR%20txid340%20%5bORGN%5d%20OR%20txid485918%20%5bORGN%5d&FIRST_QUERY_NUM=0&FORMAT_OBJECT=Alignment&FORMAT_PAGE_TARGET=&FORMAT_TYPE=HTML&GET_SEQUENCE=yes&I_THRESH=&MASK_CHAR=2&MASK_COLOR=1&NEW_VIEW=yes&NUM_OVERVIEW=100&OLD_BLAST=false&PAGE=Proteins&QUERY_INDEX=0&QUERY_NUMBER=0&RESULTS_PAGE_TARGET=&RID=GRGSF37701R&SHOW_LINKOUT=yes&SHOW_OVERVIEW=yes&STEP_NUMBER=&WORD_SIZE=3&OLD_VIEW=false&DISPLAY_SORT=2&HSP_SORT=1) | [coverage [%]](http://blast.ncbi.nlm.nih.gov/Blast.cgi?CMD=Get&ALIGNMENTS=0&ALIGNMENT_VIEW=Pairwise&CDD_SEARCH_STATE=4&DATABASE_SORT=0&DESCRIPTIONS=100&DYNAMIC_FORMAT=on&ENTREZ_QUERY=txid471854%20%5bORGN%5d%20OR%20txid62928%20%5bORGN%5d%20OR%20txid340%20%5bORGN%5d%20OR%20txid485918%20%5bORGN%5d&FIRST_QUERY_NUM=0&FORMAT_OBJECT=Alignment&FORMAT_PAGE_TARGET=&FORMAT_TYPE=HTML&GET_SEQUENCE=yes&I_THRESH=&MASK_CHAR=2&MASK_COLOR=1&NEW_VIEW=yes&NUM_OVERVIEW=100&OLD_BLAST=false&PAGE=Proteins&QUERY_INDEX=0&QUERY_NUMBER=0&RESULTS_PAGE_TARGET=&RID=GRGSF37701R&SHOW_LINKOUT=yes&SHOW_OVERVIEW=yes&STEP_NUMBER=&WORD_SIZE=3&OLD_VIEW=false&DISPLAY_SORT=4&HSP_SORT=0) | [E value](http://blast.ncbi.nlm.nih.gov/Blast.cgi?CMD=Get&ALIGNMENTS=0&ALIGNMENT_VIEW=Pairwise&CDD_SEARCH_STATE=4&DATABASE_SORT=0&DESCRIPTIONS=100&DYNAMIC_FORMAT=on&ENTREZ_QUERY=txid471854%20%5bORGN%5d%20OR%20txid62928%20%5bORGN%5d%20OR%20txid340%20%5bORGN%5d%20OR%20txid485918%20%5bORGN%5d&FIRST_QUERY_NUM=0&FORMAT_OBJECT=Alignment&FORMAT_PAGE_TARGET=&FORMAT_TYPE=HTML&GET_SEQUENCE=yes&I_THRESH=&MASK_CHAR=2&MASK_COLOR=1&NEW_VIEW=yes&NUM_OVERVIEW=100&OLD_BLAST=false&PAGE=Proteins&QUERY_INDEX=0&QUERY_NUMBER=0&RESULTS_PAGE_TARGET=&RID=GRGSF37701R&SHOW_LINKOUT=yes&SHOW_OVERVIEW=yes&STEP_NUMBER=&WORD_SIZE=3&OLD_VIEW=false&DISPLAY_SORT=0&HSP_SORT=0) | [identity[%]](http://blast.ncbi.nlm.nih.gov/Blast.cgi?CMD=Get&ALIGNMENTS=0&ALIGNMENT_VIEW=Pairwise&CDD_SEARCH_STATE=4&DATABASE_SORT=0&DESCRIPTIONS=100&DYNAMIC_FORMAT=on&ENTREZ_QUERY=txid471854%20%5bORGN%5d%20OR%20txid62928%20%5bORGN%5d%20OR%20txid340%20%5bORGN%5d%20OR%20txid485918%20%5bORGN%5d&FIRST_QUERY_NUM=0&FORMAT_OBJECT=Alignment&FORMAT_PAGE_TARGET=&FORMAT_TYPE=HTML&GET_SEQUENCE=yes&I_THRESH=&MASK_CHAR=2&MASK_COLOR=1&NEW_VIEW=yes&NUM_OVERVIEW=100&OLD_BLAST=false&PAGE=Proteins&QUERY_INDEX=0&QUERY_NUMBER=0&RESULTS_PAGE_TARGET=&RID=GRGSF37701R&SHOW_LINKOUT=yes&SHOW_OVERVIEW=yes&STEP_NUMBER=&WORD_SIZE=3&DISPLAY_SORT=3&HSP_SORT=3) | Accession number | Genelocus [Slit_] | | [Max score](http://blast.ncbi.nlm.nih.gov/Blast.cgi?CMD=Get&ALIGNMENTS=0&ALIGNMENT_VIEW=Pairwise&CDD_SEARCH_STATE=4&DATABASE_SORT=0&DESCRIPTIONS=100&DYNAMIC_FORMAT=on&ENTREZ_QUERY=txid471854%20%5bORGN%5d%20OR%20txid62928%20%5bORGN%5d%20OR%20txid340%20%5bORGN%5d%20OR%20txid485918%20%5bORGN%5d&FIRST_QUERY_NUM=0&FORMAT_OBJECT=Alignment&FORMAT_PAGE_TARGET=&FORMAT_TYPE=HTML&GET_SEQUENCE=yes&I_THRESH=&MASK_CHAR=2&MASK_COLOR=1&NEW_VIEW=yes&NUM_OVERVIEW=100&OLD_BLAST=false&PAGE=Proteins&QUERY_INDEX=0&QUERY_NUMBER=0&RESULTS_PAGE_TARGET=&RID=GRGSF37701R&SHOW_LINKOUT=yes&SHOW_OVERVIEW=yes&STEP_NUMBER=&WORD_SIZE=3&OLD_VIEW=false&DISPLAY_SORT=1&HSP_SORT=1) | [Total score](http://blast.ncbi.nlm.nih.gov/Blast.cgi?CMD=Get&ALIGNMENTS=0&ALIGNMENT_VIEW=Pairwise&CDD_SEARCH_STATE=4&DATABASE_SORT=0&DESCRIPTIONS=100&DYNAMIC_FORMAT=on&ENTREZ_QUERY=txid471854%20%5bORGN%5d%20OR%20txid62928%20%5bORGN%5d%20OR%20txid340%20%5bORGN%5d%20OR%20txid485918%20%5bORGN%5d&FIRST_QUERY_NUM=0&FORMAT_OBJECT=Alignment&FORMAT_PAGE_TARGET=&FORMAT_TYPE=HTML&GET_SEQUENCE=yes&I_THRESH=&MASK_CHAR=2&MASK_COLOR=1&NEW_VIEW=yes&NUM_OVERVIEW=100&OLD_BLAST=false&PAGE=Proteins&QUERY_INDEX=0&QUERY_NUMBER=0&RESULTS_PAGE_TARGET=&RID=GRGSF37701R&SHOW_LINKOUT=yes&SHOW_OVERVIEW=yes&STEP_NUMBER=&WORD_SIZE=3&OLD_VIEW=false&DISPLAY_SORT=2&HSP_SORT=1) | [coverage [%]](http://blast.ncbi.nlm.nih.gov/Blast.cgi?CMD=Get&ALIGNMENTS=0&ALIGNMENT_VIEW=Pairwise&CDD_SEARCH_STATE=4&DATABASE_SORT=0&DESCRIPTIONS=100&DYNAMIC_FORMAT=on&ENTREZ_QUERY=txid471854%20%5bORGN%5d%20OR%20txid62928%20%5bORGN%5d%20OR%20txid340%20%5bORGN%5d%20OR%20txid485918%20%5bORGN%5d&FIRST_QUERY_NUM=0&FORMAT_OBJECT=Alignment&FORMAT_PAGE_TARGET=&FORMAT_TYPE=HTML&GET_SEQUENCE=yes&I_THRESH=&MASK_CHAR=2&MASK_COLOR=1&NEW_VIEW=yes&NUM_OVERVIEW=100&OLD_BLAST=false&PAGE=Proteins&QUERY_INDEX=0&QUERY_NUMBER=0&RESULTS_PAGE_TARGET=&RID=GRGSF37701R&SHOW_LINKOUT=yes&SHOW_OVERVIEW=yes&STEP_NUMBER=&WORD_SIZE=3&OLD_VIEW=false&DISPLAY_SORT=4&HSP_SORT=0) | [E value](http://blast.ncbi.nlm.nih.gov/Blast.cgi?CMD=Get&ALIGNMENTS=0&ALIGNMENT_VIEW=Pairwise&CDD_SEARCH_STATE=4&DATABASE_SORT=0&DESCRIPTIONS=100&DYNAMIC_FORMAT=on&ENTREZ_QUERY=txid471854%20%5bORGN%5d%20OR%20txid62928%20%5bORGN%5d%20OR%20txid340%20%5bORGN%5d%20OR%20txid485918%20%5bORGN%5d&FIRST_QUERY_NUM=0&FORMAT_OBJECT=Alignment&FORMAT_PAGE_TARGET=&FORMAT_TYPE=HTML&GET_SEQUENCE=yes&I_THRESH=&MASK_CHAR=2&MASK_COLOR=1&NEW_VIEW=yes&NUM_OVERVIEW=100&OLD_BLAST=false&PAGE=Proteins&QUERY_INDEX=0&QUERY_NUMBER=0&RESULTS_PAGE_TARGET=&RID=GRGSF37701R&SHOW_LINKOUT=yes&SHOW_OVERVIEW=yes&STEP_NUMBER=&WORD_SIZE=3&OLD_VIEW=false&DISPLAY_SORT=0&HSP_SORT=0) | [identity[%]](http://blast.ncbi.nlm.nih.gov/Blast.cgi?CMD=Get&ALIGNMENTS=0&ALIGNMENT_VIEW=Pairwise&CDD_SEARCH_STATE=4&DATABASE_SORT=0&DESCRIPTIONS=100&DYNAMIC_FORMAT=on&ENTREZ_QUERY=txid471854%20%5bORGN%5d%20OR%20txid62928%20%5bORGN%5d%20OR%20txid340%20%5bORGN%5d%20OR%20txid485918%20%5bORGN%5d&FIRST_QUERY_NUM=0&FORMAT_OBJECT=Alignment&FORMAT_PAGE_TARGET=&FORMAT_TYPE=HTML&GET_SEQUENCE=yes&I_THRESH=&MASK_CHAR=2&MASK_COLOR=1&NEW_VIEW=yes&NUM_OVERVIEW=100&OLD_BLAST=false&PAGE=Proteins&QUERY_INDEX=0&QUERY_NUMBER=0&RESULTS_PAGE_TARGET=&RID=GRGSF37701R&SHOW_LINKOUT=yes&SHOW_OVERVIEW=yes&STEP_NUMBER=&WORD_SIZE=3&DISPLAY_SORT=3&HSP_SORT=3) | Accession number |
|  |  |  |  |  |  |  |  |  |  |  |  |  |  |  |  |  |  |  |  |  |  |  |  |  |  |  |  |  |  |  |  |  |  |  |
| 3996 | ankyrin-like protein | ankyrin-like protein | [NP_639335.1](http://www.ncbi.nlm.nih.gov/protein/21233418?report=genbank&log$=prottop&blast_rank=1&RID=GZ06Z6M7013) |  | 57.8 | 443 | 70 | 7.00E-09 | 38 | [YP_934488.1](http://www.ncbi.nlm.nih.gov/protein/119899275?report=genbank&log$=prottop&blast_rank=5&RID=GZ06Z6M7013) | | 3570 | 67.8 | 397 | 76 | 1.00E-12 | 29 | [YP_286769.1](http://www.ncbi.nlm.nih.gov/protein/71909182?report=genbank&log$=prottop&blast_rank=1&RID=YFVNAF4Y015) | | 2793 | 38.5 | 215 | 41 | 2.00E-04 | 31 | [YP_002944680.1](http://www.ncbi.nlm.nih.gov/protein/239815770?report=genbank&log$=prottop&blast_rank=11&RID=5WHX48X201R) | 392 | | 84 | 488 | 69 | 2.00E-18 | 41 | [YP_003523020.1](http://www.ncbi.nlm.nih.gov/protein/291612863?report=genbank&log$=prottop&blast_rank=1&RID=ABUGKMRG01R) |
| 3997 | hypothetical protein | hypothetical protein | [NP_639336.1](http://www.ncbi.nlm.nih.gov/protein/21233419?report=genbank&log$=prottop&blast_rank=1&RID=GZ0PAKK401R) |  |  |  |  |  |  |  | |  |  |  |  |  |  |  | |  |  |  |  |  |  |  |  | |  |  |  |  |  |  |
| 3998 | 3-oxoacyl-ACP synthase | ketosynthase | [NP_639337.1](http://www.ncbi.nlm.nih.gov/protein/21233420?report=genbank&log$=prottop&blast_rank=1&RID=GZ0VSJJK01R) | K | 365 | 365 | 99 | 3.00E-123 | 53 | [YP_935413.1](http://www.ncbi.nlm.nih.gov/protein/119900200?report=genbank&log$=prottop&blast_rank=2&RID=GZ0VSJJK01R) | | 4185 | 369 | 369 | 99 | 2.00E-125 | 50 | [YP_287381.1](http://www.ncbi.nlm.nih.gov/protein/71909794?report=genbank&log$=prottop&blast_rank=1&RID=YFVCDRXD01R) | | 262 | 489 | 489 | 100 | 9.00E-172 | 62 | [YP_002942191.1](http://www.ncbi.nlm.nih.gov/protein/239813281?report=genbank&log$=prottop&blast_rank=2&RID=5WJ50CEB01R) | 350 | | 363 | 363 | 99 | 2.00E-123 | 50 | [YP_003522979.1](http://www.ncbi.nlm.nih.gov/protein/291612822?report=genbank&log$=prottop&blast_rank=1&RID=ABUNSTZU01R) |
| 3999 | hypothetical protein | Beta-ketoacyl synthase, N-terminal domain; | [NP_639338.1](http://www.ncbi.nlm.nih.gov/protein/21233421?report=genbank&log$=prottop&blast_rank=1&RID=GZ13XBDG013) | J | 120 | 120 | 76 | 2.00E-32 | 46 | [YP_935414.1](http://www.ncbi.nlm.nih.gov/protein/119900201?report=genbank&log$=prottop&blast_rank=2&RID=GZ13XBDG013) | | 4186 | 129 | 129 | 72 | 2.00E-36 | 46 | [YP_287382.1](http://www.ncbi.nlm.nih.gov/protein/71909795?report=genbank&log$=prottop&blast_rank=1&RID=YFV7TRTS01R) | | 261 | 203 | 203 | 90 | 6.00E-64 | 53 | [YP_002942190.1](http://www.ncbi.nlm.nih.gov/protein/239813280?report=genbank&log$=prottop&blast_rank=2&RID=5WJD4KD601R) | 351 | | 115 | 115 | 79 | 2.00E-32 | 40 | [YP_003522980.1](http://www.ncbi.nlm.nih.gov/protein/291612823?report=genbank&log$=prottop&blast_rank=1&RID=ABUSA3JU01R) |
| 4000 | dolichyl-phosphate mannose synthase-like protein | glycosyltransferase | [NP_639339.1](http://www.ncbi.nlm.nih.gov/protein/21233422?report=genbank&log$=prottop&blast_rank=1&RID=GZ1FHGA801R) | E | 82 | 82 | 55 | 1.00E-18 | 34 | [YP_935419.1](http://www.ncbi.nlm.nih.gov/protein/119900206?report=genbank&log$=prottop&blast_rank=4&RID=GZ1FHGA801R) | | 4193 | 81.6 | 81.6 | 79 | 3.00E-19 | 29 | [YP_287389.1](http://www.ncbi.nlm.nih.gov/protein/71909802?report=genbank&log$=prottop&blast_rank=1&RID=YFUWS3YV01R) | | 2679 | 69.7 | 69.7 | 76 | 2.00E-14 | 29 | [YP_002944570.1](http://www.ncbi.nlm.nih.gov/protein/239815660?report=genbank&log$=prottop&blast_rank=4&RID=5WJMPH4E013) | 375 | | 74.3 | 74.3 | 76 | 7.00E-18 | 29 | [YP_003523004.1](http://www.ncbi.nlm.nih.gov/protein/291612847?report=genbank&log$=prottop&blast_rank=1&RID=ABUTWBXH01R) |
| 4001 | halogenase | halogenase | [NP_639340.1](http://www.ncbi.nlm.nih.gov/protein/21233423?report=genbank&log$=prottop&blast_rank=1&RID=GZ2103ZB013) | A | 82.8 | 82.8 | 61 | 5.00E-17 | 26 | [YP_935423.1](http://www.ncbi.nlm.nih.gov/protein/119900210?report=genbank&log$=prottop&blast_rank=3&RID=GZ2103ZB013) | | 4197 | 75.9 | 75.9 | 60 | 2.00E-15 | 24 | [YP_287393.1](http://www.ncbi.nlm.nih.gov/protein/71909806?report=genbank&log$=prottop&blast_rank=1&RID=YFUTJVSX01R) | | 260 | 587 | 587 | 99 | 0 | 55 | [YP_002942189.1](http://www.ncbi.nlm.nih.gov/protein/239813279?report=genbank&log$=prottop&blast_rank=2&RID=5WK24NFV016) | 49 | | 37 | 37 | 32 | 0.002 | 27 | [YP_003522678.1](http://www.ncbi.nlm.nih.gov/protein/291612521?report=genbank&log$=prottop&blast_rank=1&RID=ABUVB76C01R) |
| 4002 | hypothetical protein | contains ABC motiv | [NP_639341.1](http://www.ncbi.nlm.nih.gov/protein/21233424?report=genbank&log$=prottop&blast_rank=1&RID=GZ25S506013) |  |  |  |  |  |  |  | | 2559 | 36.2 | 36.2 | 44 | 0.005 | 24 | [YP_285762.1](http://www.ncbi.nlm.nih.gov/protein/71908175?report=genbank&log$=prottop&blast_rank=1&RID=YFUVHMXF01R) | | 1171 | 56.6 | 56.6 | 84 | 6.00E-09 | 24 | [YP_002943088.1](http://www.ncbi.nlm.nih.gov/protein/239814178?report=genbank&log$=prottop&blast_rank=3&RID=5WK7HCT701R) | 1168 | | 30 | 30 | 9 | 0.026 | 36 | [YP_003523793.1](http://www.ncbi.nlm.nih.gov/protein/291613636?report=genbank&log$=prottop&blast_rank=1&RID=ABUZBNZU01R) |
| 4003 | 3-ketoacyl-ACP reductase | 3-ketoacyl-ACP reductase/FabG | [NP_639342.1](http://www.ncbi.nlm.nih.gov/protein/21233425?report=genbank&log$=prottop&blast_rank=1&RID=GZ2E5NUE015) | H | 281 | 281 | 97 | 7.00E-95 | 64 | [YP_935416.1](http://www.ncbi.nlm.nih.gov/protein/119900203?report=genbank&log$=prottop&blast_rank=2&RID=GZ2E5NUE015) | | 4188 | 308 | 308 | 96 | 3.00E-106 | 68 | [YP_287384.1](http://www.ncbi.nlm.nih.gov/protein/71909797?report=genbank&log$=prottop&blast_rank=1&RID=YFUD3H6G01R) | | 2678 | 281 | 281 | 96 | 1.00E-94 | 58 | [YP_002944569.1](http://www.ncbi.nlm.nih.gov/protein/239815659?report=genbank&log$=prottop&blast_rank=4&RID=5WKAH089013) | 353 | | 323 | 323 | 96 | 4.00E-112 | 67 | [YP_003522982.1](http://www.ncbi.nlm.nih.gov/protein/291612825?report=genbank&log$=prottop&blast_rank=1&RID=ABV0W1P701R) |
| 4004 | phosphotransferase | FabA/Z like dehydratase | [NP_639343.1](http://www.ncbi.nlm.nih.gov/protein/21233426?report=genbank&log$=prottop&blast_rank=1&RID=GZ2P6WX101R) | I | 121 | 121 | 98 | 5.00E-35 | 50 | [YP_935415.1](http://www.ncbi.nlm.nih.gov/protein/119900202?report=genbank&log$=prottop&blast_rank=2&RID=5WKHNGHJ016) | | 4187 | 118 | 118 | 98 | 8.00E-35 | 47 | [YP_287383.1](http://www.ncbi.nlm.nih.gov/protein/71909796?report=genbank&log$=prottop&blast_rank=1&RID=YFU7Y37P01R) | | 2677 | 100 | 100 | 97 | 4.00E-27 | 44 | [YP_002944568.1](http://www.ncbi.nlm.nih.gov/protein/239815658?report=genbank&log$=prottop&blast_rank=4&RID=5WKHNGHJ016) | 352 | | 94 | 94 | 97 | 1.00E-25 | 46 | [YP_003522981.1](http://www.ncbi.nlm.nih.gov/protein/291612824?report=genbank&log$=prottop&blast_rank=1&RID=ABV2CE6B01R) |
| 4005 | hypothetical protein | exporter | [NP_639344.1](http://www.ncbi.nlm.nih.gov/protein/21233427?report=genbank&log$=prottop&blast_rank=1&RID=GZ2VD7FN01R) | P | 380 | 380 | 96 | 5.00E-119 | 37 | [YP_931760.1](http://www.ncbi.nlm.nih.gov/protein/119896547?report=genbank&log$=prottop&blast_rank=2&RID=GZ2VD7FN01R) | | 4177 | 413 | 413 | 96 | 3.00E-132 | 36 | [YP_287373.1](http://www.ncbi.nlm.nih.gov/protein/71909786?report=genbank&log$=prottop&blast_rank=1&RID=YFTWWFZV01R) | | 2674 | 344 | 344 | 92 | 1.00E-105 | 34 | [YP_002944565.1](http://www.ncbi.nlm.nih.gov/protein/239815655?report=genbank&log$=prottop&blast_rank=4&RID=5WKV70CS013) | 368 | | 474 | 474 | 96 | 4.00E-156 | 39 | [YP_003522997.1](http://www.ncbi.nlm.nih.gov/protein/291612840?report=genbank&log$=prottop&blast_rank=1&RID=ABV3S6J201R) |
| 4006 | hypothetical protein | hypothetical protein | [NP_639345.1](http://www.ncbi.nlm.nih.gov/protein/21233428?report=genbank&log$=prottop&blast_rank=1&RID=GZ35ACN6015) |  |  |  |  |  |  |  | |  |  |  |  |  |  |  | |  |  |  |  |  |  |  |  | |  |  |  |  |  |  |
| 4007 | fatty acyl CoA synthetase | hypothetical protein | [NP_639346.1](http://www.ncbi.nlm.nih.gov/protein/21233429?report=genbank&log$=prottop&blast_rank=1&RID=GZ372V53013) | Q | 80.9 | 80.9 | 77 | 9.00E-19 | 37 | [YP_931761.1](http://www.ncbi.nlm.nih.gov/protein/119896548?report=genbank&log$=prottop&blast_rank=2&RID=GZ372V53013) | | 4176 | 66.6 | 66.6 | 73 | 3.00E-16 | 31 | [YP_287372.1](http://www.ncbi.nlm.nih.gov/protein/71909785?report=genbank&log$=prottop&blast_rank=1&RID=YFT4PRT001R) | | 2673 | 76.3 | 76.3 | 73 | 3.00E-17 | 33 | [YP_002944564.1](http://www.ncbi.nlm.nih.gov/protein/239815654?report=genbank&log$=prottop&blast_rank=3&RID=5WKZZB0N01R) | 367 | | 61.2 | 61.2 | 81 | 6.00E-13 | 27 | [YP_003522996.1](http://www.ncbi.nlm.nih.gov/protein/291612839?report=genbank&log$=prottop&blast_rank=1&RID=ABV6REAZ01R) |
| 4008 | acyltransferase | acyltransferase | [NP_639347.1](http://www.ncbi.nlm.nih.gov/protein/21233430?report=genbank&log$=prottop&blast_rank=1&RID=GZ6KPS3G015) | R | 205 | 205 | 91 | 3.00E-63 | 40 | [YP_931762.1](http://www.ncbi.nlm.nih.gov/protein/119896549?report=genbank&log$=prottop&blast_rank=2&RID=GZ6KPS3G015) | | 4175 | 200 | 200 | 91 | 3.00E-62 | 38 | [YP_287371.1](http://www.ncbi.nlm.nih.gov/protein/71909784?report=genbank&log$=prottop&blast_rank=1&RID=YFSYGN7X01R) | | 2672 | 148 | 148 | 90 | 2.00E-41 | 36 | [YP_002944563.1](http://www.ncbi.nlm.nih.gov/protein/239815653?report=genbank&log$=prottop&blast_rank=4&RID=5WMA327E016) | 366 | | 216 | 216 | 90 | 3.00E-68 | 41 | [YP_003522995.1](http://www.ncbi.nlm.nih.gov/protein/291612838?report=genbank&log$=prottop&blast_rank=1&RID=ABV89JTE01R) |
| 4009 | dehydratase | dehydratase | [NP_639348.1](http://www.ncbi.nlm.nih.gov/protein/21233431?report=genbank&log$=prottop&blast_rank=1&RID=GZ71Z6W001R) | S | 57.8 | 57.8 | 91 | 5.00E-12 | 41 | [YP_931763.1](http://www.ncbi.nlm.nih.gov/protein/119896550?report=genbank&log$=prottop&blast_rank=2&RID=GZ71Z6W001R) | | 4174 | 54.3 | 54.3 | 91 | 1.00E-11 | 36 | [YP_287370.1](http://www.ncbi.nlm.nih.gov/protein/71909783?report=genbank&log$=prottop&blast_rank=1&RID=YFSU6YCS01R) | | 2671 | 59.7 | 59.7 | 91 | 1.00E-11 | 41 | [YP_002944562.1](http://www.ncbi.nlm.nih.gov/protein/239815652?report=genbank&log$=prottop&blast_rank=3&RID=5WMMP5C5016) | 365 | | 54.3 | 54.3 | 97 | 1.00E-11 | 31 | [YP_003522994.1](http://www.ncbi.nlm.nih.gov/protein/291612837?report=genbank&log$=prottop&blast_rank=1&RID=ABV9RF4H01R) |
| 4010 | acyltransferase | acyltransferase | [NP_639349.1](http://www.ncbi.nlm.nih.gov/protein/21233432?report=genbank&log$=prottop&blast_rank=1&RID=GZ776NW8015) |  | 83.6 | 83.6 | 69 | 3.00E-19 | 34 | [YP_932358.1](http://www.ncbi.nlm.nih.gov/protein/119897145?report=genbank&log$=prottop&blast_rank=3&RID=5WMYU8ZM016) | | 3933 | 80.1 | 80.1 | 44 | 6.00E-19 | 41 | [YP_287131.1](http://www.ncbi.nlm.nih.gov/protein/71909544?report=genbank&log$=prottop&blast_rank=1&RID=YFSP05N001R) | | 4741 | 85.5 | 85.5 | 67 | 6.00E-20 | 34 | [YP_002946612.1](http://www.ncbi.nlm.nih.gov/protein/239817702?report=genbank&log$=prottop&blast_rank=2&RID=5WMYU8ZM016) | 374 | | 55.8 | 55.8 | 57 | 2.00E-10 | 32 | [YP_003523003.1](http://www.ncbi.nlm.nih.gov/protein/291612846?report=genbank&log$=prottop&blast_rank=3&RID=ABVBCB7E01R) |
| 4011 | ketosynthase | hypothetical protein | [NP_639350.1](http://www.ncbi.nlm.nih.gov/protein/21233433?report=genbank&log$=prottop&blast_rank=1&RID=GZ7AW36Y01R) | U | 32 | 32 | 45 | 0.17 | 30 | [YP_931765.1](http://www.ncbi.nlm.nih.gov/protein/119896552?report=genbank&log$=prottop&blast_rank=2&RID=GZ7AW36Y01R) | | 4172 | 37.7 | 37.7 | 46 | 2.00E-05 | 27 | [YP_287368.1](http://www.ncbi.nlm.nih.gov/protein/71909781?report=genbank&log$=prottop&blast_rank=1&RID=YFSCABFW01R) | | 2670 | 48.9 | 48.9 | 85 | 3.00E-07 | 28 | [YP_002944561.1](http://www.ncbi.nlm.nih.gov/protein/239815651?report=genbank&log$=prottop&blast_rank=2&RID=5WN1HFXC013) | 363 | | 49.3 | 49.3 | 49 | 4.00E-09 | 28 | [YP_003522992.1](http://www.ncbi.nlm.nih.gov/protein/291612835?report=genbank&log$=prottop&blast_rank=1&RID=ABVEASRG01R) |
| 4012 | acyl carrier protein | ACP | [NP_639351.2](http://www.ncbi.nlm.nih.gov/protein/77747971?report=genbank&log$=prottop&blast_rank=1&RID=GZ7GV91K01R) | V | 90.1 | 90.1 | 83 | 2.00E-24 | 57 | [YP_931766.1](http://www.ncbi.nlm.nih.gov/protein/119896553?report=genbank&log$=prottop&blast_rank=3&RID=GZ7GV91K01R) | | 4171 | 102 | 102 | 88 | 3.00E-30 | 61 | [YP_287367.1](http://www.ncbi.nlm.nih.gov/protein/71909780?report=genbank&log$=prottop&blast_rank=1&RID=YFS42F8C01R) | | 2669 | 100 | 100 | 95 | 2.00E-28 | 59 | [YP_002944560.1](http://www.ncbi.nlm.nih.gov/protein/239815650?report=genbank&log$=prottop&blast_rank=4&RID=5WN76MA601R) | 354 | | 99.4 | 99.4 | 76 | 8.00E-29 | 59 | [YP_003522983.1](http://www.ncbi.nlm.nih.gov/protein/291612826?report=genbank&log$=prottop&blast_rank=1&RID=ABVFR4VM01R) |
| 4013 | reductase/halogenase | halogenase | [NP_639352.1](http://www.ncbi.nlm.nih.gov/protein/21233435?report=genbank&log$=prottop&blast_rank=1&RID=GZ7P5N8X01R) | A | 365 | 365 | 93 | 2.00E-123 | 46 | [YP_935423.1](http://www.ncbi.nlm.nih.gov/protein/119900210?report=genbank&log$=prottop&blast_rank=2&RID=GZ7P5N8X01R) | | 4197 | 359 | 359 | 95 | 1.00E-121 | 41 | [YP_287393.1](http://www.ncbi.nlm.nih.gov/protein/71909806?report=genbank&log$=prottop&blast_rank=1&RID=YFRS3UH101R) | | 2969 | 368 | 368 | 94 | 6.00E-123 | 44 | [YP_002944855.1](http://www.ncbi.nlm.nih.gov/protein/239815945?report=genbank&log$=prottop&blast_rank=2&RID=5WN95EWK013) |  | |  |  |  |  |  |  |
| 4014 | pteridine-dependent deoxygenase like protein | chorismatase /XanB2 | [NP_639353.2](http://www.ncbi.nlm.nih.gov/protein/77747972?report=genbank&log$=prottop&blast_rank=1&RID=GZ7X3X6B01R) | B | 154 | 154 | 89 | 6.00E-44 | 37 | [YP_935422.1](http://www.ncbi.nlm.nih.gov/protein/119900209?report=genbank&log$=prottop&blast_rank=3&RID=GZ7X3X6B01R) | | 4196 | 185 | 185 | 91 | 2.00E-56 | 38 | [YP_287392.1](http://www.ncbi.nlm.nih.gov/protein/71909805?report=genbank&log$=prottop&blast_rank=1&RID=YFRGXU7B01R) | | 2681 | 165 | 165 | 88 | 7.00E-48 | 40 | [YP_002944572.1](http://www.ncbi.nlm.nih.gov/protein/239815662?report=genbank&log$=prottop&blast_rank=4&RID=5WNDH8WJ016) | 372 | | 179 | 179 | 99 | 3.00E-54 | 37 | [YP_003523001.1](http://www.ncbi.nlm.nih.gov/protein/291612844?report=genbank&log$=prottop&blast_rank=1&RID=ABVMTTZ601R) |
| 4015 | AMP-ligase | CoA-ligase | [NP_639354.1](http://www.ncbi.nlm.nih.gov/protein/21233437?report=genbank&log$=prottop&blast_rank=1&RID=GZ84846101R) | T | 301 | 301 | 84 | 5.00E-96 | 45 | [YP_931764.1](http://www.ncbi.nlm.nih.gov/protein/119896551?report=genbank&log$=prottop&blast_rank=2&RID=GZ84846101R) | | 4173 | 300 | 300 | 83 | 4.00E-96 | 44 | [YP_287369.1](http://www.ncbi.nlm.nih.gov/protein/71909782?report=genbank&log$=prottop&blast_rank=1&RID=YFR9DNY101R) | | 2671 | 258 | 258 | 81 | 1.00E-78 | 41 | [YP_002944562.1](http://www.ncbi.nlm.nih.gov/protein/239815652?report=genbank&log$=prottop&blast_rank=4&RID=5WNFTGM9013) | 364 | | 305 | 305 | 83 | 1.00E-98 | 42 | [YP_003522993.1](http://www.ncbi.nlm.nih.gov/protein/291612836?report=genbank&log$=prottop&blast_rank=1&RID=ABVPSDBK01R) |
