# Supplementary material for: Identification and Biosynthesis of a Novel Xanthomonadin-Dialkylresorcinol-Hybrid from Azoarcus sp. BH72
Source: PLoS One. 2014 Mar 11;9(3):e90922. doi: 10.1371/journal.pone.0090922 (PMC3949708; doi:10.1371/journal.pone.0090922)
Supplement: Table S5 — Strains and plasmids used in this work. (DOCX) [file pone.0090922.s005.docx]

| Table S5. Strains and plasmids used in this work | | |
| --- | --- | --- |
| Strain or plasmid | Description | Source |
| strains |  |  |
| *E. coli* DH10B | F^–^ mcrA Δ(mrr-hsdRMS-mcrBC) Φ80lacZΔM15 ΔlacX74 recA1 endA1 araD139 Δ(ara leu) 7697 galU galK rpsL nupG λ– | [1] |
| *E. coli* BL21 (DE3) Star | F^–^ ompT hsdS_B_ (r_B_^–^ m_B_^–^) gal dcm rne131 (DE3) | Invitrogen |
| *Azoarcus* sp. BH72 | Wild type | DSMZ |
| BH0260 | *Azoarcus* sp. BH72 *azo0260*::km, km^R^ | This work |
| BH3911 | *Azoarcus* sp. BH72 *azo3911*::km, km^R^ | This work |
| TS3920 | pCATI-3920 in *E. coli* BL21 (DE3) Star, km^R^ | This work |
| TS0260 | pCATI-0260 in *E. coli* BL21 (DE3) Star, km^R^ | This work |
| plasmids |  |  |
| pCOLADuet-1 | Expressionplasmid, His_6_-Tag, km^R^ | Merck (Darmstadt) |
| pCATI1 | expression casette inserted in pCOLADuet-1, km^R^ | This work |
| pCATI-arcB | *azo3920/arcB* in pCATI1, C-terminal cherry-His_6_-tag, km^R^ | This work |
| pCATI-arcT | *azo0260/arcT* in pCATI1, C-terminal cherry-His_6_-tag, km^R^ | This work |

km^R^= kanamycine resistance

[1] Grant SG, Jessee J, Bloom FR, Hanahan D (1990) Differential plasmid rescue from transgenic mouse DNAs into Escherichia coli methylation-restriction mutants. Proc Natl Acad Sci U S A 87:4645-4649.
