# Supplementary material for: Identification and Biosynthesis of a Novel Xanthomonadin-Dialkylresorcinol-Hybrid from Azoarcus sp. BH72
Source: PLoS One. 2014 Mar 11;9(3):e90922. doi: 10.1371/journal.pone.0090922 (PMC3949708; doi:10.1371/journal.pone.0090922)
Supplement: Table S6 — Primers and PCR products used in this study. (DOCX) [file pone.0090922.s006.docx]

| Primers or PCR products | Nucleotide sequence (5´- 3´) or description* | Purpose |
| --- | --- | --- |
| primers |  |  |
| azo0260KOFor | CTGGTCTAGACCCCTCGTCACGCATACCTC | Amplification 0260KOfragment |
| azo0260KORev | GCGAAGCTTGACGATGTCCGCCGGATAGAA | Amplification 0260KOfragment |
| azo3911KOFor | CTGTCTAGACAGCCCCTTCTCCTGTCCTG | Amplification 3911KOfragment |
| azo3911KORev | GCGAAGCTTGTTCGAGCGAGTTGAAGCCG | Amplification 3911KOfragment |
| 3920fw | ATAAGGAGATATACCATGGCAACGGTTGCCGTCGTACC | Amplification 3920fragment |
| 3920rev | TTCTCGAGTGGGTTCCATCCTCGGATCGG | Amplification 3920fragment |
| *CATI3920fw* | ATGGAACCCACTCGAGAACCTATACTTC | Amplification pCATI1fragment |
| *CATI3920rev* | CCATGGTATATCTCCTTAT | Amplification pCATI1fragment |
| *0260fw* | ATAAGGAGATATACCATGGCGCTGCCCCTCGTCACGC | Amplification 0260fragment |
| *0260rev* | ATCGCTCGAGTCGCGCGCGCCGCTCCGCG | Amplification 0260fragment |
| PCR-Products |  |  |
| 3920fragment | PCR-product of *azo3920* with overlaps for pCATI1fragment | Gibson assembly pCATI-arcB |
| pCATI1fragment | PCR-product of pCATI1 with overlaps for 3920fragment | Gibson assembly pCATI-arcB |
| 0260fragment | PCR-product of *azo0260* with *Nco*I/*Xho*I restriction sites | Cloning into pCATI1 |

*recognition sites of restriction endocnucleases are underlined

Table S6
