# Supplementary material for: Identification and Biosynthesis of a Novel Xanthomonadin-Dialkylresorcinol-Hybrid from Azoarcus sp. BH72
Source: PLoS One. 2014 Mar 11;9(3):e90922. doi: 10.1371/journal.pone.0090922 (PMC3949708; doi:10.1371/journal.pone.0090922)
Supplement: Table S7 — Predicted gene clusters encoding polyene-associated biosynthesis and DAR-biosynthesis proteins. (DOCX) [file pone.0090922.s007.docx]

| **PE** | **DAR** | **class** | **order** | **family** | **strain** | **PE gene cluster** | | **DAR gene cluster** | | **gene cluster type** | **organization** |
| --- | --- | --- | --- | --- | --- | --- | --- | --- | --- | --- | --- |
|  |  |  |  |  |  | **start** | **end** | **start** | **end** |  |  |
|  |  | *Alphaproteobacteria* | *Rhodobacterales* | *Hyphomonadaceae* | *Hirschia baltica* ATCC 49814 | Hbal_1293 | Hbal_1315 | Hbal_2903 | Hbal_2895 | flexirubin | separated |
|  |  | *Betaproteobacteria* | *Burkholderiales* | *Burkholderiaceae* | *Ralstonia pickettii* 12J | Rpic_4760 | Rpic_4779 |  |  | flexirubin polyene |  |
|  |  | *Betaproteobacteria* | *Burkholderiales* | *Burkholderiaceae* | *Burkholderia ambifaria* AMMD | Bamb_3597 | Bamb_3616 |  |  | flexirubin polyene |  |
|  |  | *Betaproteobacteria* | *Burkholderiales* | *Burkholderiaceae* | *Burkholderia ambifaria* AMMD | Bamb_2821 | Bamb_2808 |  |  | xanthomonadin |  |
|  |  | *Betaproteobacteria* | *Burkholderiales* | *Burkholderiaceae* | *Burkholderia cenocepacia* HI2424 | Bcen2424_2769 | Bcen2424_2756 |  |  | xanthomonadin |  |
|  |  | *Betaproteobacteria* | *Burkholderiales* | *Comamonadaceae* | *Variovorax paradoxus* B4 | VAPA_1c28770 | VAPA_1c28640 | VAPA_1c34850 | VAPA_1c34770 | arcuflavin | separated |
|  |  | *Betaproteobacteria* | *Burkholderiales* | *Comamonadaceae* | *Variovorax paradoxus* EPS | Varpa_3239 | Varpa_3230 | Varpa_2230 | Varpa_2238 | arcuflavin | separated |
|  |  |  |  |  |  | Varpa_2705 | Varpa_2703 |  |  |  |  |
|  |  | *Betaproteobacteria* | *Burkholderiales* | *Comamonadaceae* | *Rhodoferax ferrireducens* T118 | Rfer_3980 | Rfer_4002 | Rfer_3975 | Rfer_3969 |  |  |
|  |  | *Betaproteobacteria* | *Hydrogenophilales* | *Hydrogenophilaceae* | *Thiobacillus denitrificans* ATCC 25259 | Tbd_2765 | Tbd_2742 |  |  | xanthomonadin |  |
|  |  | *Betaproteobacteria* | *Methylophilales* | *Methylophilaceae* | *Methylotenera versatilis* 301 | M301_2326 | M301_2311 |  |  | xanthomonadin |  |
|  |  | *Betaproteobacteria* | *Neisseriales* | *Neisseriaceae* | [*Pseudogulbenkiania* sp. NH8B](http://www.ncbi.nlm.nih.gov/Taxonomy/Browser/wwwtax.cgi?id=748280) | NH8B_1267 | NH8B_1246 |  |  | xanthomonadin |  |
|  |  | *Betaproteobacteria* | *Burkholderiales* | *Oxalobacteraceae* | *Herminiimonas arsenicoxydans* | HEAR2863 | HEAR2882 |  |  | xanthomonadin |  |
|  |  | *Betaproteobacteria* | *Rhodocyclales* | *Rhodocyclaceae* | *Azoarcus* sp. KH32C | AZKH_1770 | AZKH_1783 |  |  | xanthomonadin |  |
|  |  | *Betaproteobacteria* | *Rhodocyclales* | *Rhodocyclaceae* | *Aromatoleum aromaticum* EbN1 | ebA7057 | ebA7076 |  |  |  |  |
|  |  | *Betaproteobacteria* | *Burkholderiales* |  | *Thiomonas* sp. 3As | THI_2617 | THI_2599 |  |  | xanthomonadin |  |
|  |  | *Betaproteobacteria* | *Burkholderiales* |  | *Methylibium petroleiphilum* PM1 | Mpe_A1333 | Mpe_A1315 |  |  | xanthomonadin |  |
|  |  | *Cytophagia* | *Cytophagales* | *Cytophagaceae* | *Leadbetterella byssophila* DSM 17132 |  | Lbys_1472 | Lbys_1509 | Lbys_1502 | flexirubin | fused |
|  |  | *Deltaproteobacteria* | *Desulfobacterales* | *Desulfobulbaceae* | [*Desulfurivibrio alkaliphilus* AHT2](http://www.ncbi.nlm.nih.gov/Taxonomy/Browser/wwwtax.cgi?id=589865) | DaAHT2_1113 | DaAHT2_1137 | DaAHT2_1139 | DaAHT2_1144 |  | separated |
|  |  | *Deltaproteobacteria* | *Desulfobacterales* | *Desulfobulbaceae* | [*Desulfotalea psychrophila* LSv54](http://www.ncbi.nlm.nih.gov/Taxonomy/Browser/wwwtax.cgi?id=177439) | DP1860 | DP1836 |  |  |  |  |
|  |  | *Deltaproteobacteria* | *Desulfuromonadales* | *Pelobacteraceae* | [*Pelobacter carbinolicus* DSM 2380](http://www.ncbi.nlm.nih.gov/Taxonomy/Browser/wwwtax.cgi?id=338963) | Pcar_2667 | Pcar_2648 |  |  |  |  |
|  |  | *Epsilonproteobacteria* | *Campylobacterales* | *Campylobacteraceae* | *Arcobacter nitrofigilis* DSM 7299 | Arnit_2308 | Arnit_2331 | Arnit_2309 | Arnit_2312 | flexirubin | fused |
|  |  | *Epsilonproteobacteria* | *Campylobacterales* | *Campylobacteraceae* | *Sulfurospirillum barnesii* SES-3 | Sulba_2263 | Sulba_2237 | Sulba_2258 | Sulba_2255 | flexirubin | fused |
|  |  | *Epsilonproteobacteria* | *Campylobacterales* | *Campylobacteraceae* | *Sulfurospirillum deleyianum* DSM 6946 | Sdel_2097 | Sdel_2124 | Sdel_2119 | Sdel_2116 | flexirubin | fused |
|  |  | *Flavobacteriia* | *Flavobacteriales* | *Cryomorphaceae* | *Fluviicola taffensis* DSM 16823 | Fluta_1429 | Fluta_1454 | Fluta_1446 | Fluta_1451 | flexirubin | fused |
|  |  | *Flavobacteriia* | *Flavobacteriales* | *Cryomorphaceae* | *Owenweeksia hongkongensis* DSM 17368 | Oweho_1209 | Oweho_1234 | Oweho_0891 | Oweho_0881 | flexirubin | separated |
|  |  | *Flavobacteriia* | *Flavobacteriales* | *Flavobacteriaceae* | *Lacinutrix* sp. 5H-3-7-4 | Lacal_2066 | Lacal_2098 | Lacal_2073 | Lacal_2080 | flexirubin | fused |
|  |  | *Gammaproteobacteria* | *Acidithiobacillales* | *Acidithiobacillaceae* | [*Acidithiobacillus ferrivorans* SS3](http://www.ncbi.nlm.nih.gov/Taxonomy/Browser/wwwtax.cgi?id=743299) | Acife_2836 | Acife_2854 |  |  | xanthomonadin |  |
|  |  | *Gammaproteobacteria* | *Acidithiobacillales* | *Acidithiobacillaceae* | *Acidithiobacillus ferrooxidans* ATCC 53993 | Lferr_0930 | Lferr_0943 |  |  | xanthomonadin |  |
|  |  | *Gammaproteobacteria* | *Alteromonadales* |  | *Teredinibacter turnerae* T7901 | TERTU_3269 | TERTU_3289 |  |  | xanthomonadin |  |
|  |  | *Gammaproteobacteria* | *Enterobacteriales* | *Enterobacteriaceae* | *Escherichia coli* CFT073 | c1204 | c1186 |  |  |  |  |
|  |  | *Gammaproteobacteria* | *Pasteurellales* | *Pasteurellaceae* | *Actinobacillus suis* H91-0380 | ASU2_09605 | ASU2_09755 | ASU2_09630 | ASU2_09665 | flexirubin | fused |
|  |  | *Gammaproteobacteria* | *Pasteurellales* | *Pasteurellaceae* | [*Aggregatibacter aphrophilus* NJ8700](http://www.ncbi.nlm.nih.gov/Taxonomy/Browser/wwwtax.cgi?id=634176) | NT05HA_1698 | NT05HA_1718 | NT05HA_1736 | NT05HA_1744 |  | separated |
|  |  | *Gammaproteobacteria* | *Pseudomonadales* | *Pseudomonadaceae* | *Pseudomonas fulva* 12-X |  | Psefu_0467 | Psefu_0434 | Psefu_0437 | flexirubin | fused |
|  |  | *Gammaproteobacteria* | *Pseudomonadales* | *Pseudomonadaceae* | *Pseudomonas mendocina* NK-01 |  | MDS_0629 | MDS_0596 | MDS_0599 | flexirubin | fused |
|  |  | *Gammaproteobacteria* | *Vibrionales* | *Vibrionaceae* | *Aliivibrio salmonicida* LFI1238 | VSAL_I2108 | VSAL_I2090 |  |  | flexirubin polyene |  |
|  |  | *Gammaproteobacteria* | *Vibrionales* | *Vibrionaceae* | *Photobacterium profundum* SS9 | PBPRB0120 | PBPRB0104 |  |  | flexirubin polyene |  |
|  |  | *Gammaproteobacteria* | *Xanthomonadales* | *Xanthomonadaceae* | *Stenotrophomonas maltophilia* JV3 | BurJV3_3971 | BurJV3_3950 |  |  | flexirubin polyene |  |
|  |  | *Gammaproteobacteria* | *Xanthomonadales* | *Xanthomonadaceae* | *Pseudoxanthomonas spadix* BD-a59 | DSC_00380 | DSC_00460 |  |  | xanthomonadin |  |
|  |  | *Spirochaetes* | *Spirochaetales* | *Brachyspiraceae* | *Brachyspira hyodysenteriae* WA1 | BHWA1_00284 | BHWA1_00294 |  |  |  |  |

PE: polyene; DAR: dialkylresorcinol. Gene clusters were identified using primary sequences of *Azoarcus* sp. BH72 arcuflavin biosynthesis proteins (Table 1) in BLAST-P analysis with preselection for the respective class and by STRING-analysis. Identified gene clusters from bacteria listed in Table S3 and all *Xanthomonas* species are not shown. Gene clusters from the flavobacteriia and cytophagia were limited to four examples. Identified polyene-like biosynthesis gene clusters that contain a gene encoding a protein with a predicted YjgF/YER057c/UK114-like domain are labeled as xanthomonadin-type (yellow filling in first row), whereas gene clusters that contain a gene encoding a protein with a predicted PAL-HAL-domain are labeled as flexirubin polyene-type (red filling in first row). If additionally homologues of DAR biosynthesis genes were found in the genome, xanthomonadin-type gene clusters were labeled as arcuflavin-type (yellow filling in first and second row) and flexirubin polyene gene clusters were labeled as flexirubin-type (red filling in first and second row).

Table S7
