# Supplementary figures and images for: Identification and Biosynthesis of a Novel Xanthomonadin-Dialkylresorcinol-Hybrid from Azoarcus sp. BH72
Source: PLoS One. 2014 Mar 11;9(3):e90922. doi: 10.1371/journal.pone.0090922 (PMC3949708; doi:10.1371/journal.pone.0090922)

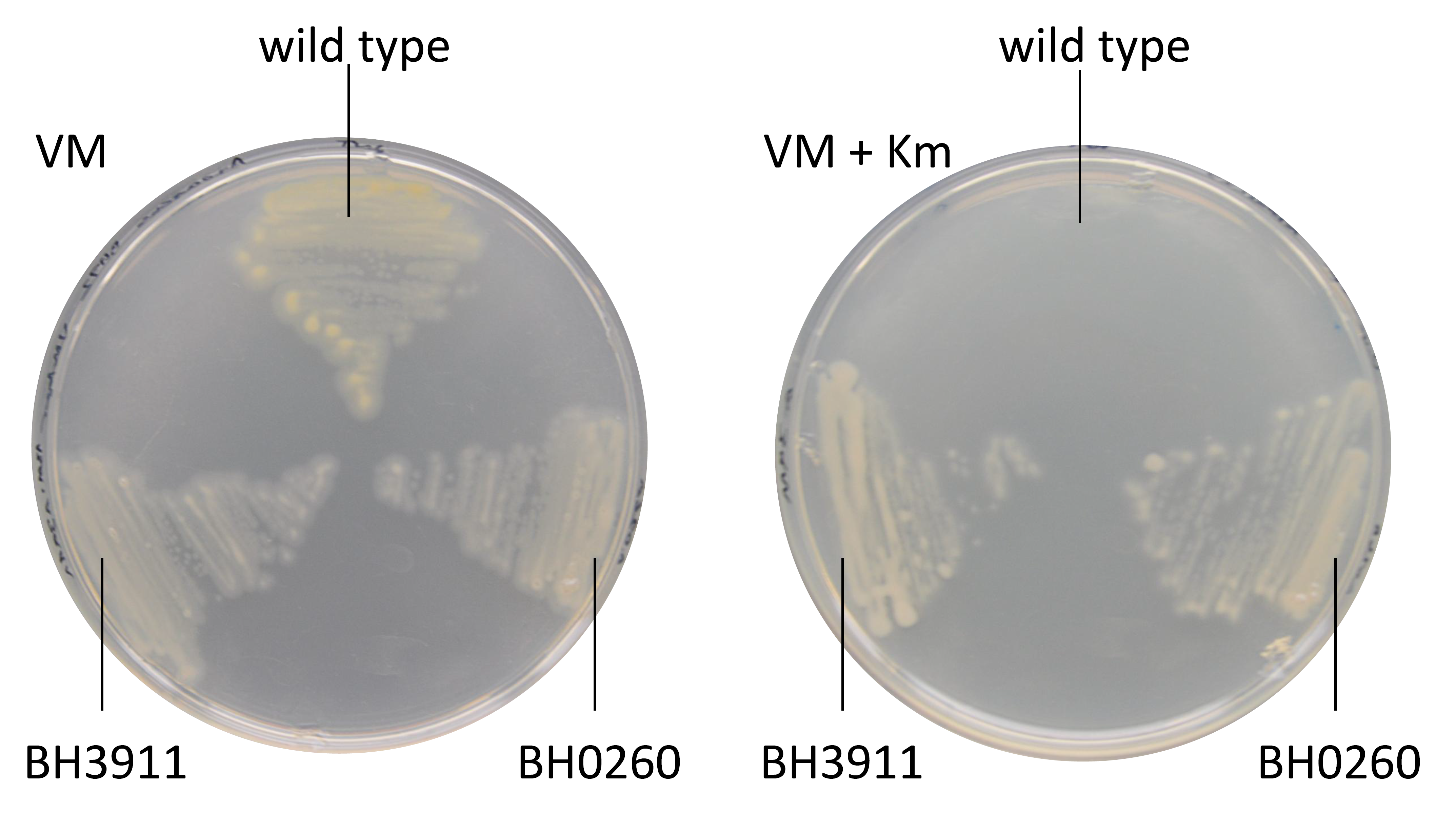

Supplement: Figure S1 — Phenotype of Azoarcus sp. BH72 wildtype and mutant strains. Growth and pigmentation of wildtype Azoarcus sp. BH72 and insertional mutants on VM-agar (with ethanol) plate (left) or a kanamycin containing VM-agar (with ethanol) plate (right). (TIF) [file pone.0090922.s008.tif]

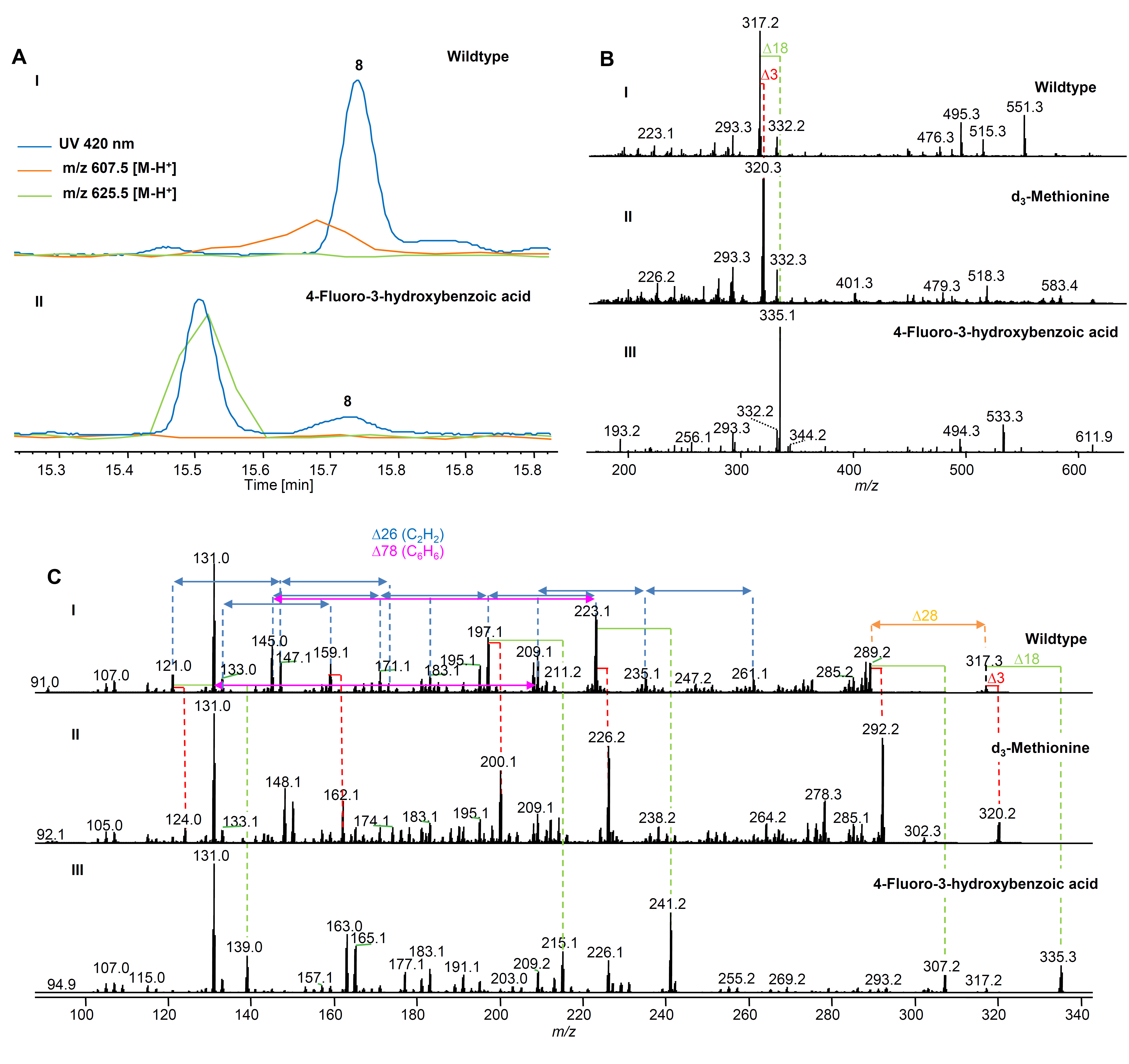

Supplement: Figure S2 — HPLC-MS-analysis and mass spectra of 8 from Azoarcus sp. BH72. A: HPLC-MS-analysis of wildtype 8 (I) and after feeding with 4-fluoro-3-hydroxybenzoic acid (4F-3HBA) (II) in raw extracts. Traces show EIC of m/z 607.5 [M-H+] (orange), m/z 625.5 [M-H+] (green) and UV at 420 nm (blue). Chromatograms are drawn to the same scale. B: MALDI-iontrap-MS2 of wildtype 8 (I), m/z 611.4 [M]+• (II) and m/z 626.4 [M]+• (III) from feeding experiments with d3-methionine or 4F-3HBA, respectively. C: MALDI-iontrap-MS3 mass spectra of the polyene-fragments m/z 317.3 (I), m/z 320.2 (II) and m/z 335.3 (III), that were obtained from the precursors m/z 608.4 [M]+•(I), m/z 611.4 [M]+• (II) and m/z 626.4 [M]+•(III). Mass shifts mentioned in the text are indicated. (TIF) [file pone.0090922.s009.tif]

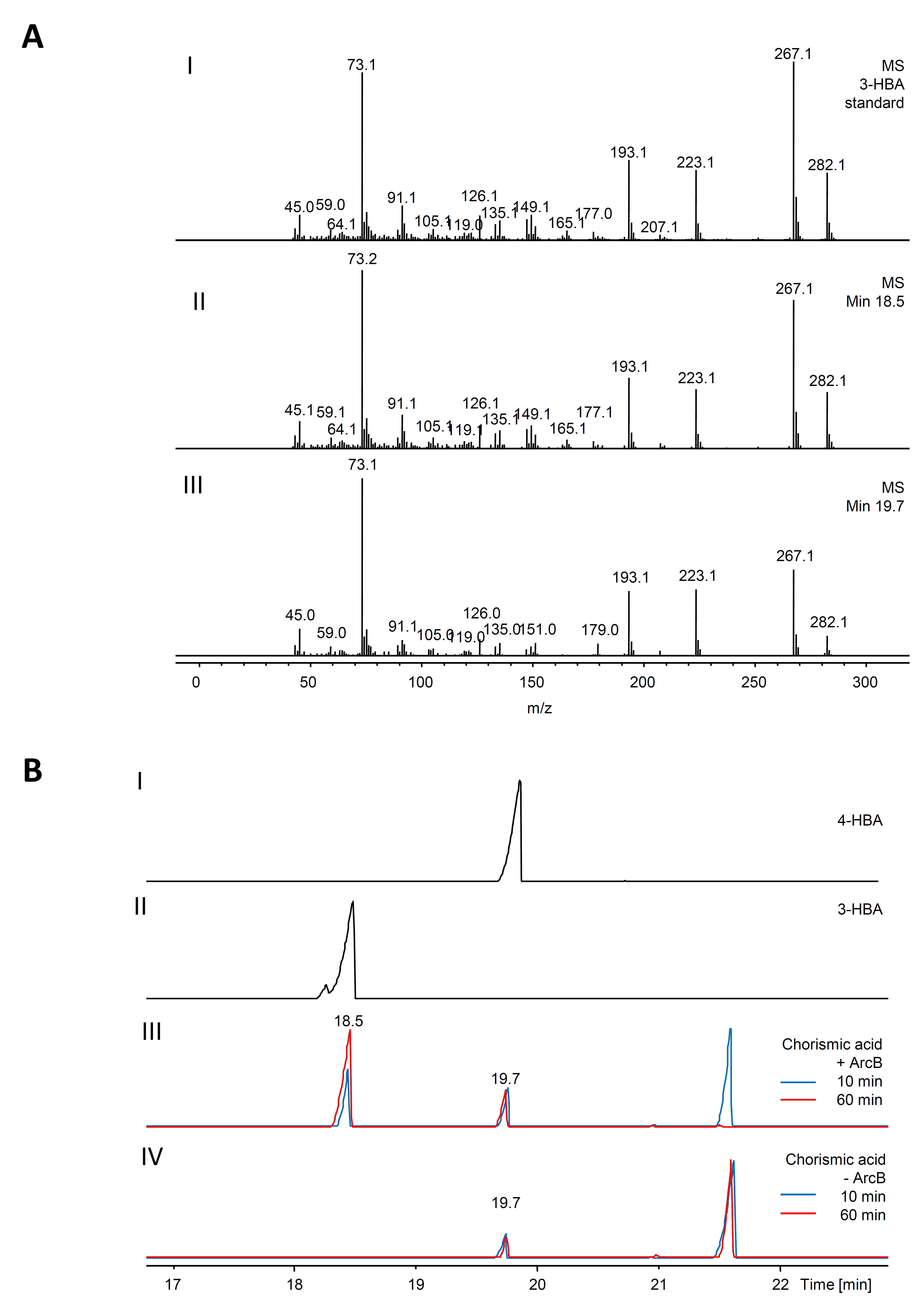

Supplement: Figure S3 — GC-MS analysis of E. coli cultures overexpressing arcB and in vitro assays with purified ArcB. A: Mass spectra of 3-hydroxybenzoic acid (3-HBA) standard (I) and the compounds identified at min 18.5 (II) and min 19.7 (III) in E. coli cultures overexpressing arcB. B: Total ion chromatograms of 4-hydroxybenzoic acid (4-HBA) standard (I) and 3-HBA standard (II) and chorismic acid containing in vitro assays incubated with (III) or without (IV) ArcB and stopped after 10 (blue) or 60 min (red) incubation, respectively. (TIF) [file pone.0090922.s010.tif]

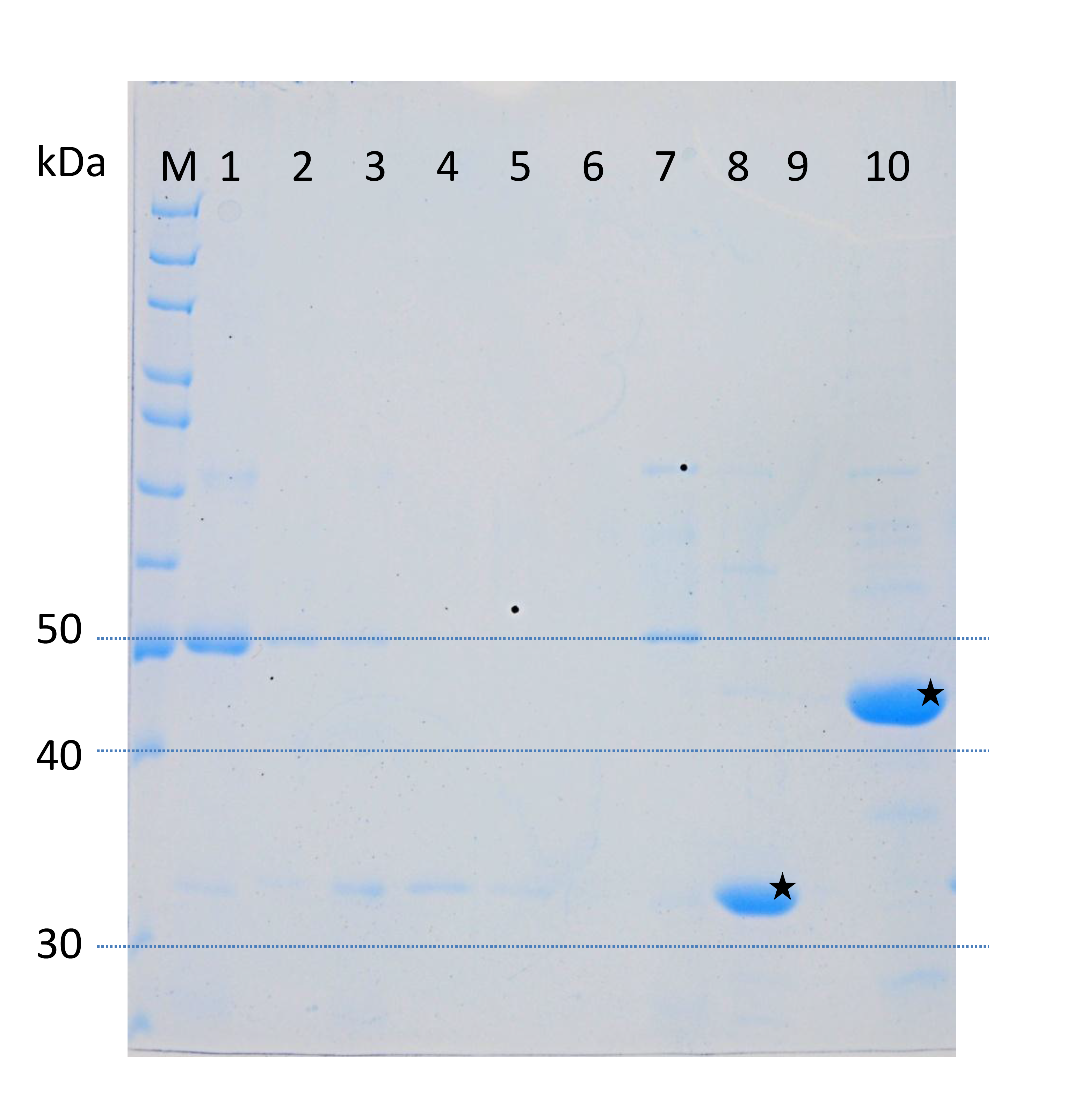

Supplement: Figure S4 — SDS-PAGE analysis of purified Azoarcus proteins. M: PageRuler™ Unstained protein ladder (Fermentas). Lane 1–8: Fractions of ArcB purification with the purified protein in Lane 8 (expected size 35,24 kDA) Lane 10: Purified ArcT (expected size: 47,1 kDa). Stars indicate protein bands with the expected size. (TIF) [file pone.0090922.s011.tif]

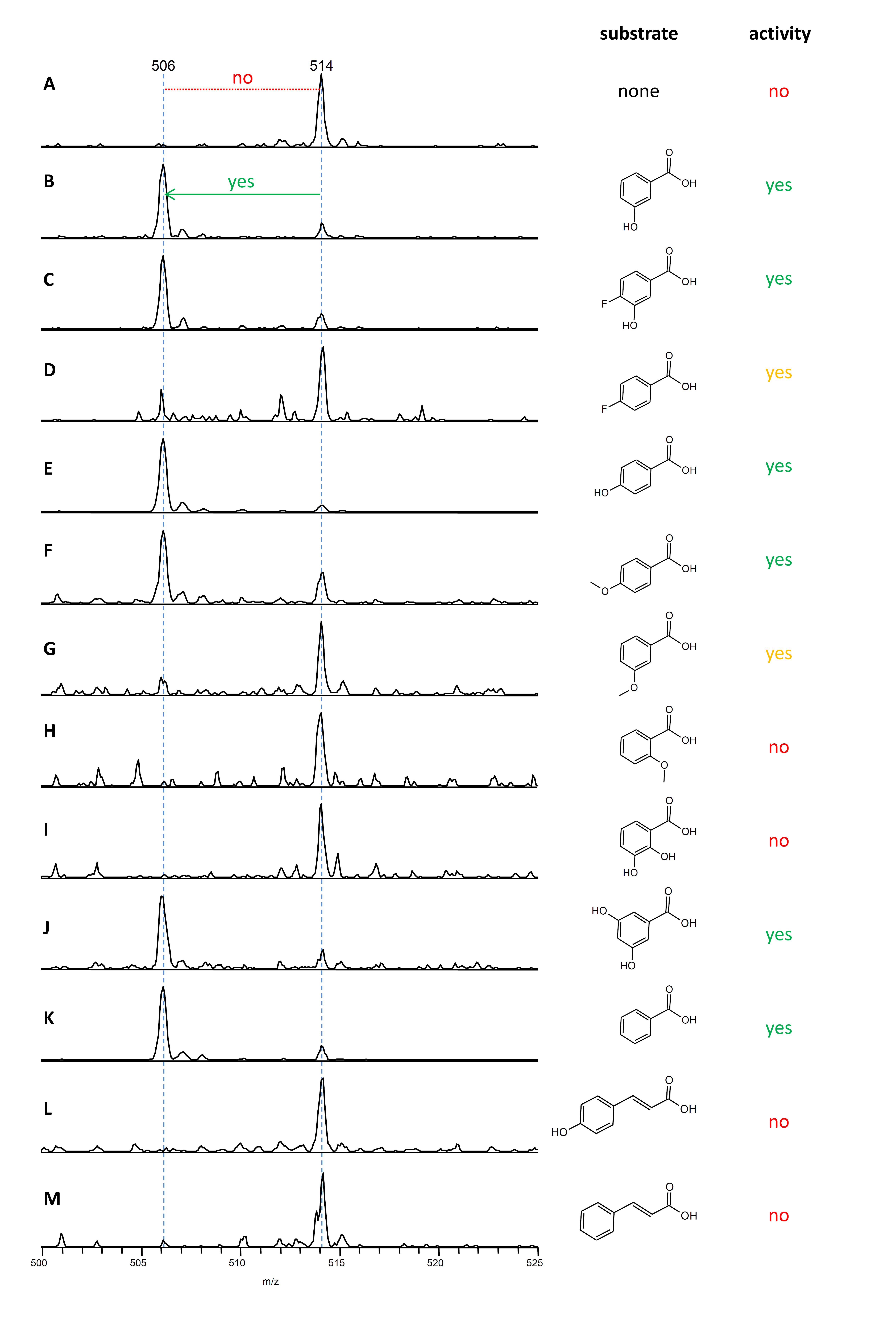

Supplement: Figure S5 — MALDI-MS analysis of [γ-18O4]-ATP containing assays with ArcT and various substrates. A mass shift from m/z 514 to m/z 506 indicates the exchange of the ATP-label and therefore activity of ArcT with the substrate. Substrates: A: none B: 3-hydroxybenzoic acid C: 4-fluoro-3-hydroxybenzoic acid D: 4-fluorobenzoic acid E: 4-hydroxybenzoic acid F: 4-methoxybenzoic acid G: 3-methoxybenzoic acid H: 2-methoxybenzoic acid I: 2,3-dihydroxybenzoic acid J: 3,5- dihydroxybenzoic acid K: benzoic acid L: p-coumaric acid M: E-cinnamic acid. Colours indicate if no (red), <50% (orange) or >50% label exchange (green) was detected. (TIF) [file pone.0090922.s012.tif]
